# Voices of patients’ relatives to support weaning from mechanical ventilation: a randomized trial

**DOI:** 10.1093/braincomms/fcaf197

**Published:** 2025-06-16

**Authors:** Maximilian I Sprügel, Marie-Louise Isenberg, Jochen A Sembill, Tamara M Welte, Rüdiger Hopfengärtner, Stefanie Balk, Kosmas Macha, Anne Mrochen, Lena Rühl, Franziska Panier, Luise Biburger, Tobias Heckelsmüller, Lisa Dietmar, Markus Prinz, Stefan Schwab, Hagen B Huttner, Joji B Kuramatsu

**Affiliations:** Universitätsklinikum Erlangen, Neurologische Klinik, 91054 Erlangen, Bayern, Germany; Universitätsklinikum Erlangen, Neurologische Klinik, 91054 Erlangen, Bayern, Germany; Universitätsklinikum Erlangen, Neurologische Klinik, 91054 Erlangen, Bayern, Germany; Universitätsklinikum Erlangen, Neurologische Klinik, 91054 Erlangen, Bayern, Germany; Universitätsklinikum Erlangen, Neurologische Klinik, 91054 Erlangen, Bayern, Germany; Universitätsklinikum Erlangen, Neurologische Klinik, 91054 Erlangen, Bayern, Germany; Universitätsklinikum Erlangen, Neurologische Klinik, 91054 Erlangen, Bayern, Germany; Universitätsklinikum Erlangen, Neurologische Klinik, 91054 Erlangen, Bayern, Germany; Universitätsklinikum Erlangen, Neurologische Klinik, 91054 Erlangen, Bayern, Germany; Universitätsklinikum Erlangen, Neurologische Klinik, 91054 Erlangen, Bayern, Germany; Universitätsklinikum Erlangen, Neurologische Klinik, 91054 Erlangen, Bayern, Germany; Universitätsklinikum Erlangen, Neurologische Klinik, 91054 Erlangen, Bayern, Germany; Universitätsklinikum Erlangen, Neurologische Klinik, 91054 Erlangen, Bayern, Germany; Universitätsklinikum Erlangen, Neurologische Klinik, 91054 Erlangen, Bayern, Germany; Universitätsklinikum Erlangen, Neurologische Klinik, 91054 Erlangen, Bayern, Germany; Universitätsklinikum Erlangen, Neurologische Klinik, 91054 Erlangen, Bayern, Germany; Universitätsklinikum Erlangen, Neurologische Klinik, 91054 Erlangen, Bayern, Germany

**Keywords:** family support, brain injuries, critical care, ventilation, weaning

## Abstract

Weaning from mechanical ventilation is complicated in patients with severe brain injury, but recurrent stimulation by familiar voices and commands to breathe in and out during critical weaning periods may improve patient outcomes. This study aimed to assess the feasibility, safety and efficacy of audio recordings of patients’ relatives to support weaning from mechanical ventilation. VOICE-WEANING II (Voices of patients’ relatives to support weaning from mechanical ventilation) was a randomized (1:1), sham-controlled clinical trial. Patients aged 18 years and older with controlled mechanical ventilation ≥ 48 h due to neurological disease were included. Patients received either audio recordings or sham control with muted audio recordings for 10 min, three times per day from initiation of assisted mechanical ventilation. The primary outcome was rate of weaning failure. Secondary outcomes included duration of controlled ventilation, rate of tracheostomy, delirium and all-cause mortality at 90 days. Brain activity was assessed using spectral density analysis of continuous electroencephalogram monitoring. Fourty-five participants were randomized (25 males/20 females, median age 60 years). Of those, 22 patients received audio recordings (48.9%) and 23 (51.1%) sham control. Rate of weaning failure was 52.4% in the intervention group and 63.6% in the control group (adjusted difference −9.5; 95% confidence interval, −38.8 to 19.9; *P* = 0.50). Duration of controlled mechanical ventilation was significantly reduced in the treatment group (adjusted difference −19.4 h; 95% confidence interval, −37.4 to −1.5 h; *P* = 0.03). The intervention was feasible and safe. Brain activity was increased in response to treatment and pronounced in right fronto-central brain regions. Although audio recordings of patients’ relatives did not significantly reduce weaning failure, the duration of controlled mechanical ventilation was significantly reduced and brain activity increased suggesting an immediate treatment response. These trial results seem to indicate a therapeutic effect of audio recordings of patients’ relatives for weaning from mechanical ventilation.

## Teaser

Sprügel *et al*. report that audio recordings reduced the duration of controlled ventilation by ∼20 h, increased brain activity in response to treatment and provide a safe, low-cost treatment option in intensive care. However, there were no statistically significant differences for the primary outcome in this early terminated trial.

## Graphical Abstract

**Graphical Abstract:**
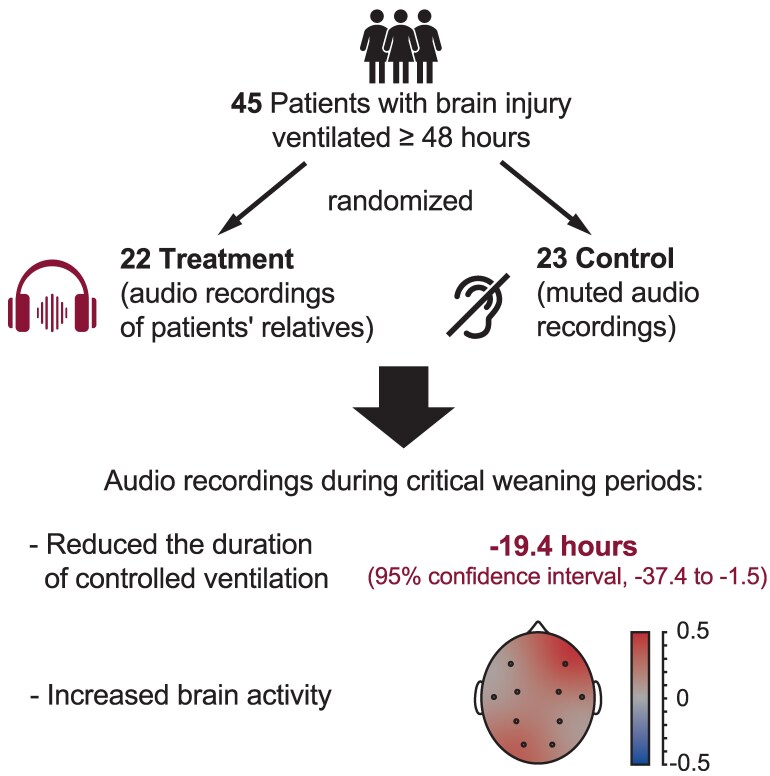

## Introduction

Patients with severe brain injuries, such as ischaemic stroke, intracranial haemorrhage, meningoencephalitis or status epilepticus, frequently require mechanical ventilation.^1-5^ However, an altered level of consciousness and depressed respiratory drive complicate weaning from ventilation.^6,7^ Notably, the transition from controlled to assisted ventilation is challenging in disoriented patients with neurological deficits. Agitation and discomfort in brain-injured patients aggravate discoordination between the patient and the ventilator resulting in a patient ‘fighting the ventilator’.^8,9^ This phenomenon causes discomfort, gas exchange deterioration and cardiovascular impairment and frequently requires deeper sedation and prolonged controlled ventilation. As a result, higher rates of weaning failure and delayed extubation lead to ventilator-associated pneumonia, necessity of tracheostomy and increased mortality.^2,4,9-11^ Therefore, improving the weaning of brain-injured patients from mechanical ventilation is warranted to prevent ventilator-associated complications and to eventually improve clinical outcomes.^10^

Intensive support by care providers including recurrent stimulation and commands to breathe in and out improves weaning of brain-injured patients and the transition from controlled to assisted ventilation, but limited resources undermine broad application in clinical routine.^12,13^ Involving family members of critically ill patients into intensive care treatment improved patient-centred care and reduced the length of stay in the intensive care unit (ICU), but implementation in clinical routine is challenging and visits of patients’ relatives were restricted during the current COVID-19 pandemic.^14-16^ Furthermore, trial interventions of family support are difficult to quantify and were not sufficiently standardized in previous studies.^17,18^ However, audio recordings of patients’ relatives (ARPR) administered during critical weaning periods represent a clearly defined trial intervention and may specifically improve weaning in brain-injured patients combining the advantages of intensive weaning support and involvement of patients’ relatives into intensive care treatment.

This randomized (1:1), sham-controlled clinical trial explored if ARPR support weaning from mechanical ventilation in patients with severe brain injury. We hypothesized that ARPR reduced the (i) rate of weaning failure, (ii) duration of controlled ventilation and (iii) rate of tracheostomy and was safe regarding (iv) rate of delirium and (v) mortality at 90 days.

## Materials and methods

### Study design and patients

We conducted a randomized clinical trial (VOICE-WEANING II) at the neurological ICU of the University Hospital Erlangen. Eligible patients had received intubation and controlled ventilation ≥ 48 h due to neurological disease; patients were not included if they were younger than 18 years of age and had history of psychiatric disease or weaning from mechanical ventilation was not intended or decision was made to limit intensive care. The trial was conducted in accordance with the International Council for Harmonisation Guideline for Good Clinical Practice and the Declaration of Helsinki and followed the Consolidated Standards of Reporting Trials (CONSORT) reporting guideline.^19,20^ The study was prospectively registered in ClinicalTrials.gov (NCT03795623). Study protocols and informed consent were approved by the local institutional review board (417_18B), and informed consent was obtained by independent study team members from legal representatives. The trial protocol is available in the Supplementary material.

### Randomization and masking

Patients were randomized with equal probabilities to the treatment group (ARPR) or the sham-control group (muted ARPR) using an online tool (www.randomizer.at/) and stratified permuted block randomization (Supplementary material ‘Methods’ section). Masking of patients was not possible owing to the intervention type. Care providers, investigators, outcome assessors and patients’ relatives were blinded to group assignment.

### Procedures

A predefined text—including information on the patient's condition and recurrent request to breathe in and out—was recorded as an audio file by one of the patient's relatives (Supplementary Figs 2 and 3). Audio recordings were conducted at our ICU in a dedicated quite room by an independent study team member with the selected person of the patient's relatives. Voice recordings averaged 1 min and were transferred to a secure server. Audio editing was performed by the independent study team member unblinded to randomization using an open-source audio software (www.audacityteam.org/) to reduce the dynamic range, to insert an initial audio notification (request to put the headphones on the patient’s head) and to create ARPR of ∼10-min length by repeating the voice recordings with audio breaks of 0.25 min in between. For the sham-control group, ARPR were muted resulting in mute audio recordings without audio signals except for the initial audio notification. This independent study team member transferred the audio recordings to a portable media player.

Audio recordings were administered when the first conversion from controlled to assisted mechanical ventilation was performed according to current guideline recommendations.^21^ Thereafter, audio recordings were administered three times per day (in the morning between 6:00 and 8:00; at midday between 12:00 and 14:00 and in the evening between 18:00 and 20:00), but only during assisted mechanical ventilation. Spontaneous breathing trials (SBTs), when intended according to current guideline recommendations, were performed during administration of the audio recordings. Readiness to wean was assessed using criteria according to current guidelines adapted to neurological patients (Supplementary Table 4). Initiation of weaning was defined as the time point when initial dose reduction of sedative medications was performed after clinical decision was made to initiate weaning.

### Outcomes

The primary outcome was the rate of weaning failure defined as reintubation and/or resumption of ventilatory support within 48 h following extubation or death within 48 h following extubation or failed SBT (for criteria of failed SBT, see Supplementary Table 5).^21^ Secondary efficacy outcomes were duration of controlled mechanical ventilation after weaning initiation and rate of tracheostomy within 28 days after start of ventilation or discharge from intensive care, whichever came first. Secondary safety outcomes were rate of ICU delirium within 28 days after start of ventilation or discharge from intensive care, whichever came first, and rate of all-cause mortality at 90 days after start of ventilation. Tracheostomy was defined as surgically created airway by open surgical or percutaneous dilation technique.^22,23^ Delirium was monitored according to the Confusion Assessment Method for ICU.^24^

### Statistical analysis

Statistical analyses were performed using SPSS version 24 and STATA 17.0. Analysis method of primary and secondary outcomes was modified intention to treat; randomized participants receiving at least 1 administration of study procedure were included. Two-sided statistical tests were performed with a significance level at *α* = 0.05. Categorical variables are presented as number and percentages, compared by the Pearsons *χ*^2^ test or the Fisher’s exact test, as appropriate. Absolute differences were calculated using Wilson procedure without a correction for continuity as measure of effect. Ordinal and non-normally distributed continuous variables are presented as median and interquartile ranges (IQRs), compared by the Mann–Whitney *U*-test. Normally distributed continuous variables are compared by the *t*-test.

*Post hoc* statistical adjustments for confounder variables were performed after sensitivity analyses to address bias due to limited patient numbers after early trial termination. Confounders were identified using standardized mean differences for parameters in inter-group comparison and for parameters associated with each of the investigated primary and secondary outcomes (Supplementary material ‘Methods’ section). Individual patient data of the VOICE-WEANING I and VOICE-WEANING II were pooled for exploratory subgroup analyses. Multivariable logistic regression models were constructed using the same methodology as aforementioned. Exploratory power spectral density analysis of continuous EEG monitoring data was performed, and power values of frequency bands were normalized to individual participant level (IPL; Supplementary material ‘Methods’ section).

Sample size was calculated with 80% power and 5% α-risk for the hypothesis of ARPR achieving a 15% absolute decrease in weaning failure on the basis of an event rate of 44% in the sham-control group.^25^ The sample size was increased by 10% to correct for dropouts and lost to follow-up resulting in 354 patients. An interim analysis was planned after inclusion of 50% of the calculated subjects.

## Results

Prior to VOICE WEANING II, we conducted a proof-of-concept study, VOICE-WEANING I, to evaluate potential treatment effects of ARPR using a single-centre, non-randomized pre/post-intervention design (see Supplementary material for further details). Among 43 patients, 23 in the control group and 20 patients in the treatment group, ARPR reduced the duration of controlled mechanical ventilation [ARPR: median 24.4; IQR, 11.1–35.8 h; control: median 46.1; IQR, 22.9–75.3 h; adjusted difference −20.4 h; 95% confidence interval (CI), −39.5 to −1.2 h; *P* = 0.04; Supplementary Tables 1 and 2 and Supplementary Fig. 1] suggesting a therapeutic effect of ARPR for weaning from mechanical ventilation. These data served as the basis for initiation and design of the VOICE-WEANING II trial.

**Figure 1:**
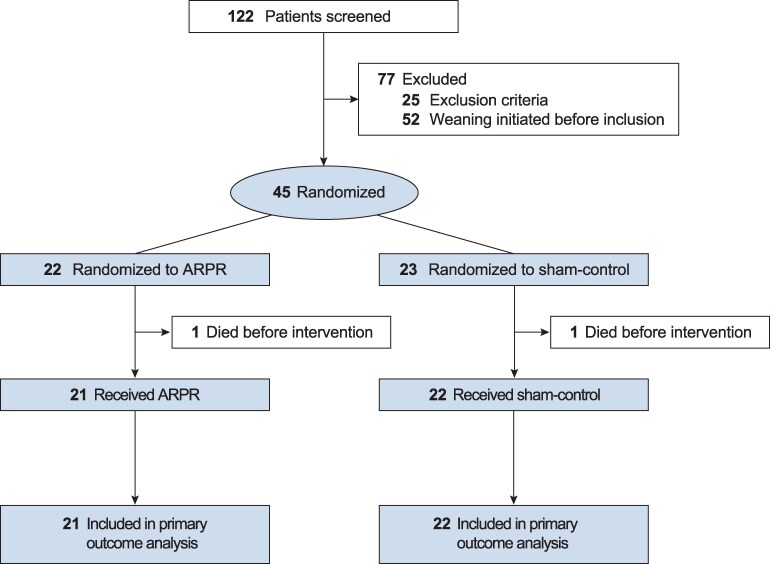
**Flow of patients in the VOICE-WEANING II randomized clinical trial.** ARPR, audio recordings of patients’ relatives.

Between 1 February 2019 and 31 December 2020, 122 patients were screened, of whom 77 did not meet eligibility criteria, resulting in 45 patients recruited in VOICE-WEANING II (Fig. 1). Since March 2020, enrolment to the trial was temporarily halted due to the COVID-19 pandemic, and in January 2021, the trial was terminated early owing to ongoing COVID-19 hospital visiting restrictions, not allowing to record voices of patients’ relatives according to the trial protocol, which resulted in delayed recruitment and consequently expiration of the funding period. At the time that the trial was halted, 13% of the anticipated number of patients had been enrolled; 22 patients were randomized to the treatment arm and 23 patients to the sham-control arm; 43 patients received at least one administration of their randomized intervention [21 ARPR (95.5%); 22 sham control (95.7%)]. All patients completed the 3-month follow-up period. Participant demographic and disease characteristics among intervention groups appeared globally balanced and were not significantly different (Table 1; Supplementary Table 3). The median age was 60 (IQR, 53–68) years; participants were ventilated for a median duration of 5.3 (IQR, 3.3–8.0) days prior to weaning initiation and 11.9 (IQR, 9.9–16.2) days overall. Tracheostomy was performed a median of 9.1 (IQR, 6.5–10.7) days after initiation of ventilation, and the most frequent admission diagnosis was intracerebral haemorrhage (34.9%). The median one-way road travel distance between hospital and home of patient’s relatives was 38.4 (IQR, 17.6–57.8) km, and the median two-way travel duration was 1.2 (IQR, 0.7–1.7) h by car.

**Table 1:** Characteristics of the trial cohort

Characteristics	ARPR (*n* = 21)	Control (*n* = 22)	Unadjusted difference (95% CI)	SMD
Age, years, median (IQR)	62 (54–71)	58 (51–65)	4 (−4 to 11)	0.32
Female patients, *n* (%)	9 (42.9)	10 (45.5)	−2.6 (−32.3 to 27.1)	−0.05
BMI, kg/m^2^, median (IQR)	26.3 (24.8–30.8)	27.7 (23.4–32.3)	−1.4 (−5.9 to 3.1)	−0.22
Patient tobacco use, *n* (%)				
Never	8 (38.1)	8 (36.4)	1.7 (−27.2 to 30.6)	0.03
Current	9 (42.9)	9 (40.9)	1.9 (27.5 to 31.4)	0.04
Previous	4 (19.0)	5 (22.7)	−3.7 (−27.9 to 20.6)	−0.09
Duration of ventilation prior to randomization, days, median (IQR)	4.0 (2.8–5.6)	4.1 (3.0–7.0)	−0.1 (−2.3 to 2.1)	−0.18
Duration of ventilation prior to weaning initiation, days, median (IQR)	5.3 (2.8–7.5)	5.4 (3.7–8.7)	−0.1 (−2.4 to 2.3)	−0.02
Mode of ventilation prior to weaning initiation, *n* (%)				
Pressure-controlled ventilation, *n* (%)	21 (100)	22 (100)	0 (0–0)	0.00
Respiratory measures prior to weaning initiation, median (IQR)				
Tidal volume, ml/kg PBW^a^	7.8 (6.6–8.1)	7.8 (6.6–8.1)	0.04 (−0.8 to 0.9)	0.27
PEEP, cmH_2_O	7 (5–9)	7 (5–9)	0 (−2 to 2)	−0.04
Respiratory rate, /min	15 (13–16)	16 (14–17)	−1 (−2 to 1)	−0.27
FiO_2_	0.35 (0.30–0.40)	0.35 (0.30–0.40)	0.0 (−0.05 to 0.05)	−0.03
PaO_2_/FiO_2_, mmHg	276 (163–306)	259 (220–316)	26 (−47 to 99)	0.02
PaCO_2_, mmHg	42.2 (39.2–47.7)	44.4 (40.0–47.9)	−2.0 (7.5 to 3.5)	−0.03
Admission diagnosis, *n* (%)				
Ischaemic stroke	6 (28.6)	6 (27.3)	1.3 (−25.5 to 28.1)	0.03
Intracerebral haemorrhage	8 (38.1)	7 (31.8)	6.3 (−22.2 to 34.7)	0.13
Subarachnoid haemorrhage	5 (23.8)	9 (40.9)	−17.1 (−44.6 to 10.4)	−0.36
Other^b^	2 (9.5)	0 (0.0)	9.5 (−0.3 to 22.1)	0.45
GCS before intubation, median (IQR)^c^	10 (7–14)	11 (8–15)	−1 (−6 to 4)	−0.25

ARPR, audio recordings of patients’ relatives; BMI, body mass index (weight in kilograms divided by height in meters squared); FiO_2_, inspiratory oxygen fraction; GCS, Glasgow Coma Scale; IQR, interquartile range; PaO_2_, arterial oxygen tension; PaCO_2_; arterial carbon dioxide tension; PBW, predicted body weight; SMD, standardized mean difference.

^a^PBW was calculated as 50 + 0.91 × [height (cm) − 152.4] for men and 45.5 + 0.91 × [height (cm) − 152.4] for women.

^b^The underlying diseases of two patients were classified as other diseases that both were status epilepticus.

^c^GCS score ranges from 3 to 15, with higher values indicating greater level of consciousness.

### Primary outcome

The primary outcome, rate of weaning failure, was reduced, but no significant difference was observed between treatment groups [ARPR: 11/21 (52.4%) versus control: 14/22 (63.6%); unadjusted difference −9.5; 95% CI, −38.8 to 19.9; adjusted difference −11.3; 95% CI, −37.3 to 17.0; *P* = 0.50; Table 2]. Failed SBT accounted for 10 of 11 (90.9%) weaning failure in the ARPR group and 11 of 14 (78.6%) in the control group (Supplementary Table 6). There was no evidence of heterogeneity of treatment effect among patient subgroups (Supplementary Fig. 4).

**Table 2:** Clinical outcomes

Outcomes	ARPR (*n* = 21)	Control (*n* = 22)	Unadjusted difference (95% CI)	Adjusted difference (95% CI)	*P*-value
Primary					
Weaning failure, *n* (%)^a^	11/21 (52.4)	14/22 (63.6)	−11.3 (−37.3 to 17.0)	−9.5 (−38.8 to 19.9)	0.50
Secondary efficacy					
Duration of controlled mechanical ventilation after weaning initiation, hours, median (IQR)^b^	22.5 (13.3–37.5)	44.2 (29.9–64.2)	−22.3 (−43.3 to −1.5)	−19.4 (−37.4 to −1.5)	**0**.**03**
Tracheostomy, *n* (%)^c^	9/21 (42.9)	11/22 (50.0)	−7.1 (−33.8 to 21.1)	−11.6 (−35.6 to 12.3)	0.34
Secondary safety					
ICU delirium, *n* (%)^d^	12/21 (57.1)	13/22 (59.1)	−2.0 (−29.1 to 25.5)	−7.0 (−36.5 to 22.5)	0.64
All-cause mortality at 90 days, *n* (%)^e^	3/21 (14.3)	4/22 (18.2)	−3.9 (−26.3 to 19.2)	−5.8 (−28.3 to 16.7)	0.62

ARPR, audio recordings of patients’ relatives; ICU, intensive care unit; IQR, interquartile range; CI, confidence interval; SBT, spontaneous breathing trial; SD, standard deviation. *P* values refer to adjusted outcome analyses (*post hoc*). Bold values represent statistically significant results (*P* < 0.05).

^a^Weaning failure was defined as reintubation and/or resumption of ventilatory support within 48 h following extubation or death within 48 h following extubation or failed SBT (for criteria of failed SBT, see Supplementary Table 4).^22^ Differences were adjusted for age, admission diagnosis (confounders identified in inter-group comparison), PaO_2_/FiO_2_ prior to weaning initiation and GCS before intubation (outcome-specific confounders). Unadjusted *P*-value: 0.46.

^b^Differences in controlled mechanical ventilation after weaning initiation were adjusted for age, admission diagnosis (confounders identified in inter-group comparison), duration of ventilation prior to weaning initiation and PaO_2_/FiO_2_ prior to weaning initiation (outcome-specific confounders). Unadjusted *P*-value: 0.01.

^c^Tracheostomy was defined as surgically created airway by open surgical or percutaneous dilation technique.^5,23,24^ Differences were adjusted for age, admission diagnosis (confounders identified in inter-group comparison), duration of ventilation prior to weaning initiation and GCS before intubation (outcome-specific confounders). Unadjusted *P*-value: 0.64.

^d^ICU delirium was defined according to the Confusion Assessment Method for ICU 25. Differences were adjusted for age, admission diagnosis (confounders identified in inter-group comparison), respiratory rate prior to weaning initiation and PaO_2_/FiO_2_ prior to weaning initiation (outcome-specific confounders). Unadjusted *P*-value: 0.90.

^e^Differences in all-cause mortality at 90 days were adjusted for age, admission diagnosis (confounders identified in inter-group comparison), patient tobacco use and PEEP prior to weaning initiation (outcome-specific confounders). Unadjusted *P*-value: 0.73.

### Secondary outcomes

Compared with control, ARPR significantly reduced the duration of controlled mechanical ventilation by 19.4 h (ARPR: median 22.5; IQR, 13.3–37.5 h; control: median 44.2; IQR 29.9–64.2 h; adjusted difference −19.4 h; 95% CI, −37.4 to −1.5 h; *P* = 0.03). Rate of tracheostomy was 42.9% in the treatment group and 50.0% in the control group (adjusted difference −11.6; 95% CI, −35.6 to 12.3; *P* = 0.34). Rate of all-cause mortality at 90 days was 14.3% in the treatment group and 18.2% in the control group (adjusted difference −5.8, 95% CI, −28.3 to 16.7; *P* = 0.62). Rate of ICU delirium did not differ across intervention groups [ARPR: 12/21 (57.1%) versus control: 13/22 (59.1%); adjusted difference −7.0, 95% CI −36.5 to 22.5; *P* = 0.64].

### Exploratory subgroup analyses

For exploratory subgroup analysis, we pooled individual patient data of VOICE-WEANING I and VOICE-WEANING II to assess the treatment effect of ARPR on the duration of controlled ventilation after weaning initiation. Overall, there were 81 patients, 39 (48.1%) in the treatment group and 42 (51.9%) in the control group. After adjustment for age, gender, body mass index, tobacco use, PaO_2_/FiO_2_ ratio, admission diagnosis and Glasgow Coma Scale before intubation, the adjusted difference in duration of controlled ventilation was −20.5 (95% CI, −33.7 to −7.4) h. Treatment effects of ARPR were not different across subgroups (Supplementary Fig. 5).

### Exploratory EEG analyses

**Figure 2:**
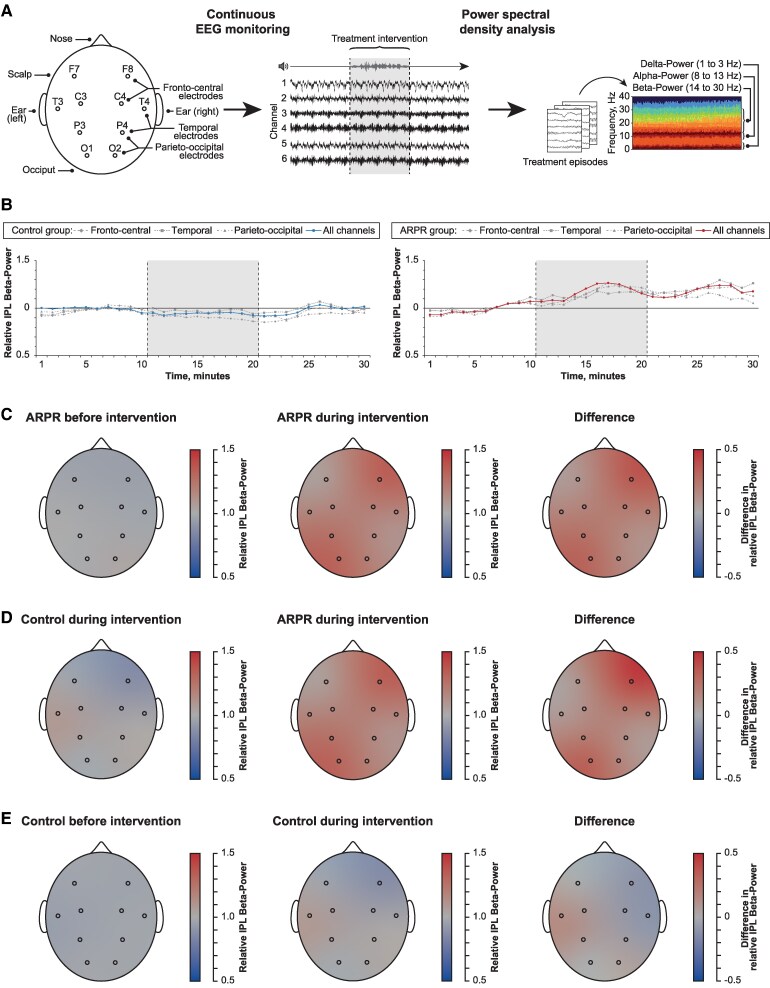
**Temporal and regional patterns of brain activity in response to treatment.** Exploratory EEG analyses were performed to assess brain activity as an immediate physiological response to ARPR. (**A**) EEG data were derived from fronto-central (F8-C4), temporal (T4-P4) and parieto-occipital (P4-O2) channels by continuous EEG monitoring performed in the ICU. EEG power values of frequency bands were calculated using power spectral density analysis and normalized to IPL values (for details, see Supplementary material ‘Methods’ section). (**B**) Temporal patterns of relative IPL beta-power values, an indicator of brain activity: before, during (grey area) and after treatment intervention for the control group (left, *N* = 12) and the ARPR group (right, *N* = 11). Method of moving average was applied to correct for overestimation. (**C**) Regional patterns of relative IPL beta-power values in the ARPR group before treatment intervention (left, *N* = 11), during intervention (middle, *N* = 11) and for the difference between during and before intervention (right, *N* = 11; numbers represent mean differences, F8-C4: 0.36; 95% CI, 0.10–0.62; *P* = 0.01; *t* = 3.13 using paired samples *t*-test). (**D**) Regional patterns of relative IPL beta-power values in the control group during treatment intervention (left, *N* = 12), in the ARPR group during intervention (middle, *N* = 11) and for the difference between ARPR and control group during intervention (right, *N* = 23; numbers represent mean differences, F8-C4: 0.44; 95% CI, 0.19–0.69; *P* = 0.001; *t* = 3.72 using independent samples *t*-test). (**E**) Regional patterns of relative IPL beta-power values in the control group before treatment intervention (left, *N* = 12), during intervention (middle, *N* = 12) and for the difference between during and before intervention (right, *N* = 12; numbers represent mean differences, T3-P3: 0.14; 95% CI, −0.09 to 0.36; *P* = 0.21; *t* = 1.33 using paired samples *t*-test). ARPR, audio recordings of patients’ relatives; C3 and C4, central scalp electrode in Positions 3 and 4, respectively, according to the 10–20 system; EEG, electroencephalogram; F7 and F8, frontal scalp electrode in Positions 7 and 8, respectively; IPL, individual participant level; O1 and O2, occipital scalp electrode in Positions 1 and 2, respectively; P3 and P4, parietal scalp electrode in Positions 3 and 4, respectively; T3 and T4; temporal electrode in Positions 3 and 4, respectively.

Exploratory power spectral density analysis of continuous EEG monitoring was performed to assess brain activity as an immediate physiological response to ARPR (Fig. 2A). Overall, there were 23 treatment interventions of 15 patient with high-quality EEG data, 11 (47.8%) in the ARPR group and 12 (52.2%) in the control group. Brain activity gradually increased during treatment interventions in the ARPR group (relative IPL beta-power before ARPR: median 0.97, IQR 0.91–1.02; during ARPR: median 1.22, IQR 1.08–1.30; *P* = 0.01), but not in response to sham interventions in the control group (Fig. 2B). Brain activation was pronounced in the right fronto-central brain region in response to ARPR in the treatment group and in inter-group comparison, but not in the control group (Fig. 2C–E). Analysis of alpha–delta ratio yielded similar results (Supplementary Fig. 6).

## Discussion

To our knowledge, this represents the first randomized clinical trial investigating the effect of ARPR to support weaning from mechanical ventilation. Regarding the primary outcome, we did not observe a significant difference in the rate of weaning failure between the two groups. Yet, the duration of controlled mechanical ventilation was significantly reduced in the intervention group, increased brain activity was detected as an immediate physiological response to ARPR, and there were no safety concerns related to the intervention. This trial confirms the results of our proof-of-concept study and suggests beneficial effects of ARPR on weaning from mechanical ventilation in patients with severe brain injury.

Regarding treatment efficacy, there was a numerical reduction of weaning failure by 11.3% in the ARPR group, slightly lower than anticipated by prior sample size calculation. This difference was not statistically significant, but the trial was terminated early and therefore not powered to show a difference for the primary endpoint. However, participants in the ARPR group had a significantly reduced duration of controlled ventilation by ∼20 h. As prolonged ventilation, notably in controlled ventilation mode, is associated with ventilator-associated complications and worse outcomes, ARPR may reduce the risk of ventilator-associated complications and may translate into improved clinical outcomes among patients with severe brain injury.^2,4,9,10,26,27^

ARPR offers manifold advantages for implementation of family support in the ICU. We here demonstrate that recording voices of patients’ relatives is feasible to overcome geographical distances (median travel time between hospital and home of patient’s relatives was 1.2 h in the VOICE WEANING II trial) and to ensure that patients may perceive familiar voices of their relatives during critical weaning periods. Furthermore, standardization of the procedure in our trial addressed major shortcomings of previous studies investigating family involvement in critical care.^17,18^ ARPR may also attenuate social isolation and visiting restrictions of patients isolated for infection control precautions to prevent transmission of multi-resistant organisms or infectious diseases during pandemics.^15,28^

ARPR provides a low-cost individual treatment option for patients in the ICU. Costs of technical equipment including recording device, headphones, audio player and randomization tool were well below 50€ per patient in this trial. Given the technical specifications of modern mobile devices and utilization of open-source software, costs of ARPR might even be lower in clinical routine. In contrast, daily costs of an ICU day range between ∼3000 and 10 000€ and costs of standard treatment interventions range between ∼500€ (invasive blood pressure monitoring) and 3000€ (tracheostomy).^29-31^

The VOICE WEANING study results indicate that ARPR represents a safe and well-tolerated treatment intervention. Rate of delirium was not increased in the intervention group. Considering recent research findings that family involvement is beneficial in prevention and treatment of delirium, ARPR appears safe and may even exert beneficial effects.^32,33^ Several studies currently evaluate music interventions and virtual reality simulations, partly combined with voices of patients’ relatives, to reduce the incidence of delirium in critically ill patients.^34-36^ However, these treatment interventions are applied during fixed time points (as compared with critical periods in our trial), not aimed at the improvement of weaning from mechanical ventilation and not focused on patients with brain injury.

Furthermore, we demonstrate increased brain activity in response to ARPR treatment using power spectral density analysis of continuous EEG monitoring in this study. Activation was detected in the whole brain but was more pronounced in right frontal brain regions that play an integral role for recognition of familiar voices and processing of their acoustic information.^37-40^ Increased brain activity reported in this study should primarily be interpreted as immediate physiological response to acoustic stimulation by familiar voices, although increased brain activity was also reported to be associated with weaning success in a previous study among brain-injured patients.^41^

Weaning from mechanical ventilation is complicated in patients with critical illness apart from primary brain injury, and these patients might also benefit from ARPR. Besides effects on internal organ function, critical illness occasionally exerts deleterious effects on the central nervous system leading to brain dysfunction and impaired consciousness.^42,43^ Notably, these patients with secondary brain injury should be included in further studies evaluating ARPR in patients with critical illness apart from primary brain injury.

### Limitations

This study has several limitations. First of all, the trial was terminated early owing to the COVID-19-pandemic-related visiting restrictions, delayed recruitment and expiration of the funding period. Therefore, the trial was not powered to show a difference in the primary outcome and uncertainty remains regarding the results of secondary outcomes and regarding subgroup analyses. Although care providers, outcome assessors and patients’ relatives were blinded to group assignment, the intervention type prevented masking of patients. Furthermore, this is a single-centre trial, which limits generalizability. Future studies might focus on patients that already tolerate spontaneous ventilation modes to avoid confounding by acute complications of critical illness. Hearing loss should be more precisely defined in the inclusion/exclusion criteria of future studies, and auditory-evoked potentials could be performed among eligible patients with increased risk of profound hearing loss. Preparation of audio recordings and administration of ARPR were elaborate and time-consuming in this trial. These were conducted exclusively by in-person visits at our ICU in a dedicated quite room. Emotional stress of patients’ relatives generally required several attempts to achieve appropriate voice recordings, and audio editing comprised different steps before and after randomization. This process should be optimized by automated audio recording and editing using app-based devices to facilitate ease of recording in a familiar environment with reduced emotional stress, to improve recruitment and to ensure feasibility of ARPR in future multicentre trials.

## Conclusion

Among brain-injured patients requiring controlled mechanical ventilation ≥ 48 h, the duration of controlled ventilation was reduced by ∼20 h in the ARPR group. The intervention would provide a safe, well-tolerated and low-cost individual treatment option implementing family support in the ICU. Future evaluation of ARPR-based family involvement is warranted in the broad spectrum of intensive care patients.

## Supplementary Material

fcaf197_Supplementary_Data

## Data Availability

The authors confirm that the data supporting the findings of this study are available within the article and its Supplementary material. Raw data are available from the corresponding author for independent scientific research upon reasonable request.
