# Supplementary material for: Voices of patients’ relatives to support weaning from mechanical ventilation: a randomized trial
Source: Brain Commun. 2025 Jun 16;7(3):fcaf197. doi: 10.1093/braincomms/fcaf197 (PMC12168123; doi:10.1093/braincomms/fcaf197)
Supplement: fcaf197_Supplementary_Data [file fcaf197_supplementary_data.docx]

**Voices of patients' relatives to support weaning from mechanical ventilation: a randomized trial**

Maximilian I. Sprügel; Marie-Louise Isenberg; Jochen A. Sembill; Tamara M. Welte; Rüdiger Hopfengärtner; Stefanie Balk; Kosmas Macha; Anne Mrochen; Lena Rühl; Franziska Panier; Luise Biburger; Tobias Heckelsmüller; Lisa Dietmar; Markus Prinz; Stefan Schwab; Hagen B. Huttner; Joji B. Kuramatsu

**Online Supplementary Material**

**Supplementary Methods:** The VOICE-WEANING I study

**Study Design and Participants**

VOICE-WEANING I is a proof of concept study conducted at the University of Erlangen-Nürnberg, a single tertiary care center and comprised patients with primary spontaneous intracerebral hemorrhage (ICH), 18 years or older and mechanical ventilation ≥48 hours in whom weaning from mechanical ventilation was intended according to standard treatment using a non-randomised pre/post intervention design. ICH patients admitted to the neurological intensive care unit between July 1, 2015 and December 31, 2015 were included into the control group, patients admitted between January 1, 2016 and June 30, 2016 were included into the treatment group. The study were approved by the local institutional review board and consent was obtained by patients or legal representatives.

**Study Procedure**

Audio recordings of patients relatives (ARPR) were recorded, edited and administered as described in the methods section of the main manuscript.

**Outcomes**

The primary outcome in VOICE-WEANING I was duration of controlled mechanical ventilation. Secondary outcomes were rate of tracheotomy within 28 days after start of ventilation or discharge from intensive care, whichever came first; rate of all-cause mortality at 90 days after start of ventilation; rate of ICU delirium within 28 days after start of ventilation or discharge from intensive care, whichever came first; and rate of weaning failure within 28 days after start of ventilation, or until 48 hours after extubation or discharge from intensive care, whichever came first. Delirium was defined according to the Confusion Assessment Method for intensive care unit (CAM-ICU) ([1](#_ENREF_1)). Weaning failure was defined as reintubation and/or resumption of ventilatory support within 48 hours following extubation or death within 48 hours following extubation or failed SBT (for criteria of failed SBT see eTable 4) ([2](#_ENREF_2)).

**Statistical Analysis**

Two-sided statistical tests were performed with a significance level at α=0.05. Categorial variables are presented as number and percentages, compared by the Pearsons χ2 test or the Fisher’s exact test, as appropriate. Absolute differences were used as measure of effect. Ordinal and non-normally distributed continuous variables are presented as median and interquartile ranges, compared by the Mann–Whitney U-test. Normally distributed continuous variables are compared by the t-test.

Confounders were identified using standardized mean differences (SMD) for parameters in intergroup comparison and for parameters associated with each investigated outcome. Median split was conducted for continuous outcome variables (i.e. duration of controlled mechanical ventilation after weaning initiation). Logistic regression models (augmented inverse probability weighting) were adjusted for relevant confounder variables (SMD>0.3) with the number of accounted variables being limited to one confounder variable per 10 patients included in respective outcome analyzes. Therefore, outcome analyzes were adjusted for age, gender, PaO_2_/FiO_2_ prior to weaning initiation (confounders identified in inter-group comparison; eTable 1) and additionally for one outcome-specific confounder: Patient tobacco use (duration of controlled mechanical ventilation after weaning initiation); duration of ventilation prior to weaning initiation (tracheotomy); PEEP prior to weaning initiation (all-cause mortality at 90 days); GCS before intubation (ICU delirium); PaO_2_/FiO_2_ prior to weaning initiation (analysis of weaning failure).

**Results**

Between July 1, 2015 and December 31, 2015, we recruited 23 patients in the control-group, and between January 1, 2016 and June 30, 2016 we recruited 20 patients in the treatment group of VOICE-WEANING I (non-randomised pre/post intervention study; eFigure 1). In total, 5 patients were excluded (3 because of weaning initiated after <48 hours of mechanical ventilation and 2 because of weaning not initiated) and 37 (18 ARPR [100%], 19 control [95.0%]) patients completed the 3 months follow-up period. Participant demographic and disease characteristics were comparable across groups (eTable 1).

Regarding the primary outcome, duration of controlled mechanical ventilation, the treatment group required less controlled ventilation (ARPR: median, 24.4 hours; range, 11.1-35.8; control: median, 46.1 hours; range, 22.9-75.3; unadjusted absolute difference [UAD] -25.2; 95% CI, -48.8 to -1.5; adjusted absolute difference [AAD] -20.4; 95% CI, -39.5 to -1.2; p=0.04; eTable 2).

Rate of tracheostomy was 50.0% in the treatment group and 50.0% in the control group (UAD 0.0; 95% CI, -29.0 to 29.0; AAD 7.9; 95% CI, -24.5 to 40.3; p=0.63). Rate of all-cause mortality at 90 days was 16.7% in the treatment group and 21.1% in the control group (UAD -4.4; 95% CI, -29.2 to 21.4; AAD -4.1; 95% CI, -26.5 to 18.2; p=0.72). Rate of ICU-delirium did not differ across intervention groups (ARPR: 7/18[38.9%] vs control: 8/20[40.0%]; UAD -1.1; 95% CI, -29.4 to 27.8; AAD -0.8; 95% CI, -32.4 to 27.9; p=0.88). Rate of weaning failure was 44.4% in the treatment group and 60.0% in the control group (UAD -15.6; 95% CI, -42.5 to 15.0; AAD -9.1; 95% CI, -39.6 to 21.5; p=0.51).

**Supplemental Figure 1:** Flow of Patients in the VOICE-WEANING I non-randomised pre/post intervention study


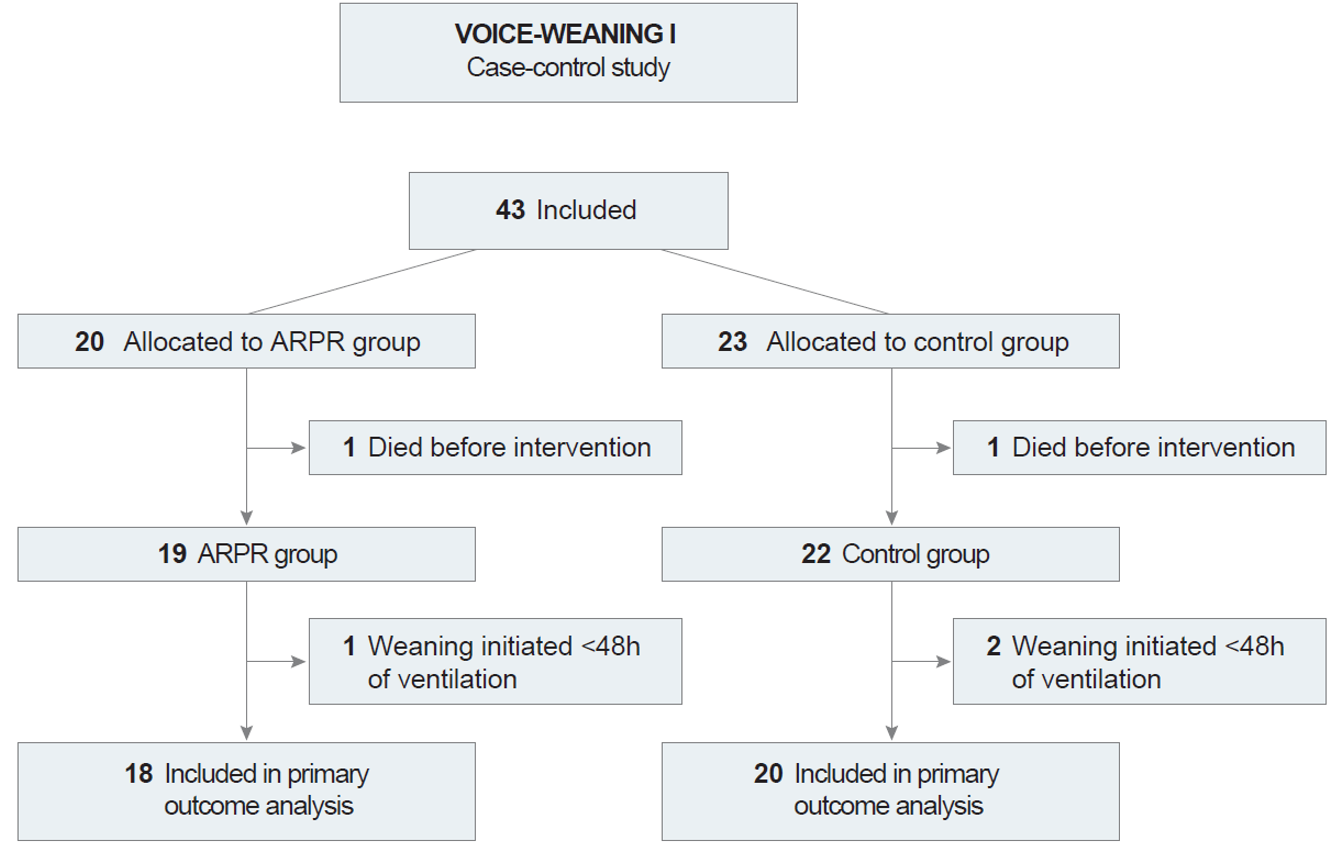


| **Characteristics** | **ARPR**  **(n=18)** | **Control**  **(n=20)** | **p value** | **SMD** |
| --- | --- | --- | --- | --- |
| Age, median (IQR), years | 64 (50-76) | 68 (60-74) | 0.57 | -0.32 |
| Female patients, n. (%) | 7 (38.9%) | 10 (50.0%) | 0.49 | -0.22 |
| BMI, median (IQR) | 28.8  (26.0-31.2) | 27.6  (25.5-30.4) | 0.65 | 0.06 |
| Patient tobacco use, n. (%) |  |  |  |  |
| Never | 11 (61.1%) | 12 (60.0%) | 0.94 | 0.02 |
| Current | 4 (22.2%) | 4 (20.0%) | 1.00 | 0.05 |
| Previous | 3 (16.7%) | 4 (20.0%) | 1.00 | -0.08 |
| Duration of ventilation prior to  weaning initiation, median (IQR), days | 3.0  (2.6-4.1) | 3.1  (2.5-5.9) | 0.85 | -0.08 |
| Mode of ventilation prior to  weaning initiation, n. (%) |  |  |  |  |
| Pressure-controlled ventilation | 18 (100%) | 20 (100%) | 1.00 | 0.00 |
| Respiratory measures prior to  weaning initiation, median (IQR) |  |  |  |  |
| Tidal volume, ml/kg PBW | 7.3  (6.3-8.8) | 7.5  (6.5-9.1) | 0.70 | -0.12 |
| PEEP, cm H_2_O | 6 (5-8) | 6 (5-8) | 0.90 | -0.05 |
| Respiratory rate, /min | 15 (13-16) | 16 (13-17) | 0.39 | -0.19 |
| FiO_2_, median (IQR) | 0.35  (0.30-0.40) | 0.35  (0.30-0.40) | 0.85 | -0.06 |
| PaO_2_ / FiO_2_, median (IQR), mm Hg | 252  (210-308) | 306  (239-329) | 0.23 | -0.23 |
| PaCO_2_, median (IQR), mm Hg | 41.9  (35.8-45.9) | 42.1  (35.7-45.0) | 0.72 | 0.19 |
| GCS before intubation, median (IQR)^b^ | 7 (3-10) | 7 (3-12) | 0.61 | -0.14 |

**Supplemental Table 1: Characteristics of the VOICE-WEANING I cohort**

Abbreviations: ARPR, audio recordings of patients' relatives; BMI, body mass index (weight in kilograms divided by height in meters squared); FiO_2_, inspiratory oxygen fraction; GCS, Glasgow Coma Scale; ICH, intracerebral hemorrhage, IVH, intraventricular hemorrhage; IQR, interquartile range; PaO_2_, arterial oxygen tension; PaCO_2_; arterial carbon dioxide tension; PBW, predicted body weight; SMD, standardized mean difference. ^a^ PBW was calculated as 50 + 0.91 × (height [cm] − 152.4) for men and 45.5 + 0.91 × (height [cm] − 152.4) for women. ^b^ GCS score ranges from 3 to 15, with higher values indicating greater level of consciousness.

| **Outcomes** | **ARPR**  **(n=18)** | **Control**  **(n=20)** | **Unadjusted**  **difference**  **(95% CI)** | **Adjusted**  **difference**  **(95% CI)** | **P value** |
| --- | --- | --- | --- | --- | --- |
| **Primary** |  |  |  |  |  |
| Duration of controlled mechanical ventilation after weaning initiation, median (IQR), hours ^a^ | 24.4  (11.1-35.8) | 46.1  (22.9-75.3) | -25.2  (-48.8 to -1.5) | - 20.4  (-39.5 to -1.2) | 0.04 |
| **Secondary** |  |  |  |  |  |
| Tracheotomy, n. (%) ^b^ | 9/18  (50.0%) | 10/20  (50.0%) | 0.0  (-29.0 to 29.0) | 7.9  (-24.5 to 40.3) | 0.63 |
| All-cause mortality at 90 days, n. (%) ^c^ | 3/18  (16.7%) | 4/19  (21.1%) | -4.4  (-29.2 to 21.4) | -4.1  (-26.5 to 18.2) | 0.72 |
| ICU delirium, n. (%) ^d^ | 7/18  (38.9%) | 8/20  (40.0%) | -1.1  (-29.4 to 27.8) | -0.8  (-32.4 to 27.9) | 0.88 |
| Weaning failure, n. (%) ^e^ | 8/18  (44.4%) | 12/20  (60.0%) | -15.6  (-42.5 to 15.0) | -9.1  (-39.6 to 21.5) | 0.51 |

**Supplemental Table 2: Clinical Outcomes of the VOICE-WEANING I study**

Abbreviations: ARPR, audio recordings of patients' relatives; ICU, intensive care unit; IQR, interquartile range; CI, confidence interval; SBT, spontaneous breathing trial; SD, standard deviation. P values refer to adjusted outcome analyses.

^a^ Differences in controlled mechanical ventilation after weaning initiation were adjusted for age, gender, PaO_2_/FiO_2_ prior to weaning initiation (confounders identified in inter-group comparison) and patient tobacco use (outcome-specific confounder). Unadjusted P value: <.01.

^b^ Tracheotomy was defined as surgically created airway by open surgical or percutaneous dilation technique. Differences were adjusted for age, gender, PaO_2_/FiO_2_ prior to weaning initiation (confounders identified in inter-group comparison) and duration of ventilation prior to weaning initiation (outcome-specific confounder). Unadjusted P value: 1.00.

^c^ Differences in all-cause mortality at 90 days were adjusted for age, gender, PaO_2_/FiO_2_ prior to weaning initiation (confounders identified in inter-group comparison) and PEEP prior to weaning initiation (outcome-specific confounder). Unadjusted P value: 1.00.

^d^ ICU delirium was defined according to the Confusion Assessment Method for intensive care unit (CAM-ICU). Differences were adjusted for age, gender, PaO_2_/FiO_2_ prior to weaning initiation (confounders identified in inter-group comparison) and GCS before intubation (outcome-specific confounder). Unadjusted P value: .94.

^e^ Weaning failure was defined as reintubation and/or resumption of ventilatory support within 48 hours following extubation or death within 48 hours following extubation or failed SBT (for criteria of failed SBT see eTable 4). Differences were adjusted for age, gender, PaO_2_/FiO_2_ prior to weaning initiation (confounders identified in inter-group comparison) and PaO_2_/FiO_2_ prior to weaning initiation (outcome-specific confounder). Unadjusted P value: .34.

**Supplementary Methods:** The VOICE-WEANING II study

**Randomization**

Randomization was generated in permuted blocks and stratified for disease entity (ischemic stroke, intracerebral hemorrhage, subarachnoid hemorrhage and other diseases), age (≤60 years and >60years) and Glasgow Coma Scale (GCS) before intubation (3-4, 5-12, 13-15) ([3](#_ENREF_3), [4](#_ENREF_4)) .

**Statistical Analysis**

Post hoc statistical adjustments for confounder variables were performed after sensitivity analyses to address bias due to limited patient numbers after early trial termination. Confounders were identified using standardized mean differences (SMD) for parameters in intergroup comparison and for parameters associated with each of the investigated primary and secondary outcomes. Median split was conducted for continuous outcome variables (i.e. duration of controlled mechanical ventilation after weaning initiation). For multivariable logistic regression models we used a doubly-robust methodology (augmented inverse probability weighting) to calculate adjusted absolute treatment effects, adjusted for relevant confounder variables (SMD>0.3) with the number of adjustment-variables being limited to one confounder variable per ten patients included in respective outcome analyzes. Therefore, outcome analyzes were adjusted for age and admission diagnosis (confounders identified in intergroup comparison; Table 1) and additionally for two outcome-specific confounders: PaO2/FiO2 prior to weaning initiation and GCS before intubation (analysis of weaning failure); duration of ventilation prior to weaning initiation and PaO2/FiO2 prior to weaning initiation (duration of controlled mechanical ventilation after weaning initiation); duration of ventilation prior to weaning initiation and GCS before intubation (tracheostomy); respiratory rate prior to weaning initiation and PaO2/FiO2 prior to weaning initiation (ICU delirium); patient tobacco use and PEEP prior to weaning initiation (all-cause mortality at 90 days).

Individual patient data of the VOICE-WEANING I and VOICE-WEANING II were pooled for exploratory subgroup analyzes to assess the treatment effect of ARPR on duration of controlled ventilation after weaning initiation among specific patient subgroups. Multivariable logistic regression models were constructed using the same methodology as aforementioned, adjusted for age, gender, tobacco use, PaO2/FiO2 prior to weaning initiation, admission diagnosis (confounders identified in inter-group comparison) and additionally body mass index and GCS before intubation (outcome-specific confounders).

Bedside EEG monitoring was performed in routine ICU clinical practice. Analyses were restricted to a simplified 10-electrode EEG montage (C3/C4, P3/P4, O2/O1, T3/T4 and F7/F8) according to the international 10/20 system ([5-7](#_ENREF_5)). We applied a high-pass filter of 0.05 Hz and a low-pass filter of 100 Hz (Carefusion EEG-system; Natus Mediacal Inc), an automated artefact rejection and bipolar montage (fronto-central channels: F8-C4; temporal channels: T4-P4; parieto-occipital channels: P4-O2) ([8](#_ENREF_8)). 30-minute EEG episodes (10-minute period before treatment initiation, 10-minute period during treatment intervention and 10-minute period after treatment) underwent power spectral density (PSD) analysis; raw EEG signals were subdivided in 2-second EEG epochs with Fourier transformation applied to determine 1-minute power values of different frequency bands (relative delta-power: 0.5 to 3.5 Hz; relative theta-power: 4-7.5 Hz; relative alpha-power: 8 to 12 Hz; relative beta-power: 12.5 to 22 Hz). Relative power was defined as the proportion of the global spectrum and alpha-delta ratio as alpha-power divided by beta-power. Power values of frequency bands were normalized to individual participant level (IPL) to account for inter-individual differences; every 1-minute power value was divided by the mean of this power value over the 10-minute time period before treatment initiation. Power values before treatment intervention were defined as the mean of this power value over a 5-minute time period (from 10 minutes before treatment initiation until 5 minutes before treatment initiation) and power values during treatment intervention were defined as the mean of this power value over a 5-minute time period (from 5 minutes after treatment initiation until 10 minutes after treatment initiation) to ensure sufficient temporal distance and identify potential effects of the treatment intervention.

| **Characteristics** | **ARPR**  **(n=21)** | **Control**  **(n=22)** |
| --- | --- | --- |
| Prior comorbidities, n. (%) |  |  |
| Hypertension | 16 (76.2%) | 17 (77.3%) |
| Diabetes | 3 (14.3%) | 5 (22.7%) |
| Prior ischemic stroke or TIA | 3 (14.3%) | 3 (13.6%) |
| Prior hemorrhagic stroke or major bleeding | 1 (4.8%) | 0 (0.0%) |
| Congestive heart failure | 0 (0.0%) | 2 (9.1%) |
| Coronary artery disease | 2 (9.5%) | 4 (18.2%) |
| Prior myocardial infarction | 0 (0.0%) | 2 (9.1%) |
| Atrial fibrillation | 2 (9.5%) | 1 (4.5%) |
| Peripheral artery disease | 1 (4.8%) | 0 (0.0%) |
| Chronic obstructive pulmonary disease | 2 (9.5%) | 0 (0.0%) |

**Supplemental Table 3: Additional Characteristics of the VOICE-WEANING II cohort**

Abbreviations: ARPR, audio recordings of patients' relatives; TIA, transient ischemic attack.

**Supplemental Figure 2: Predefined text recorded as an audio file by one of the patient's relatives**


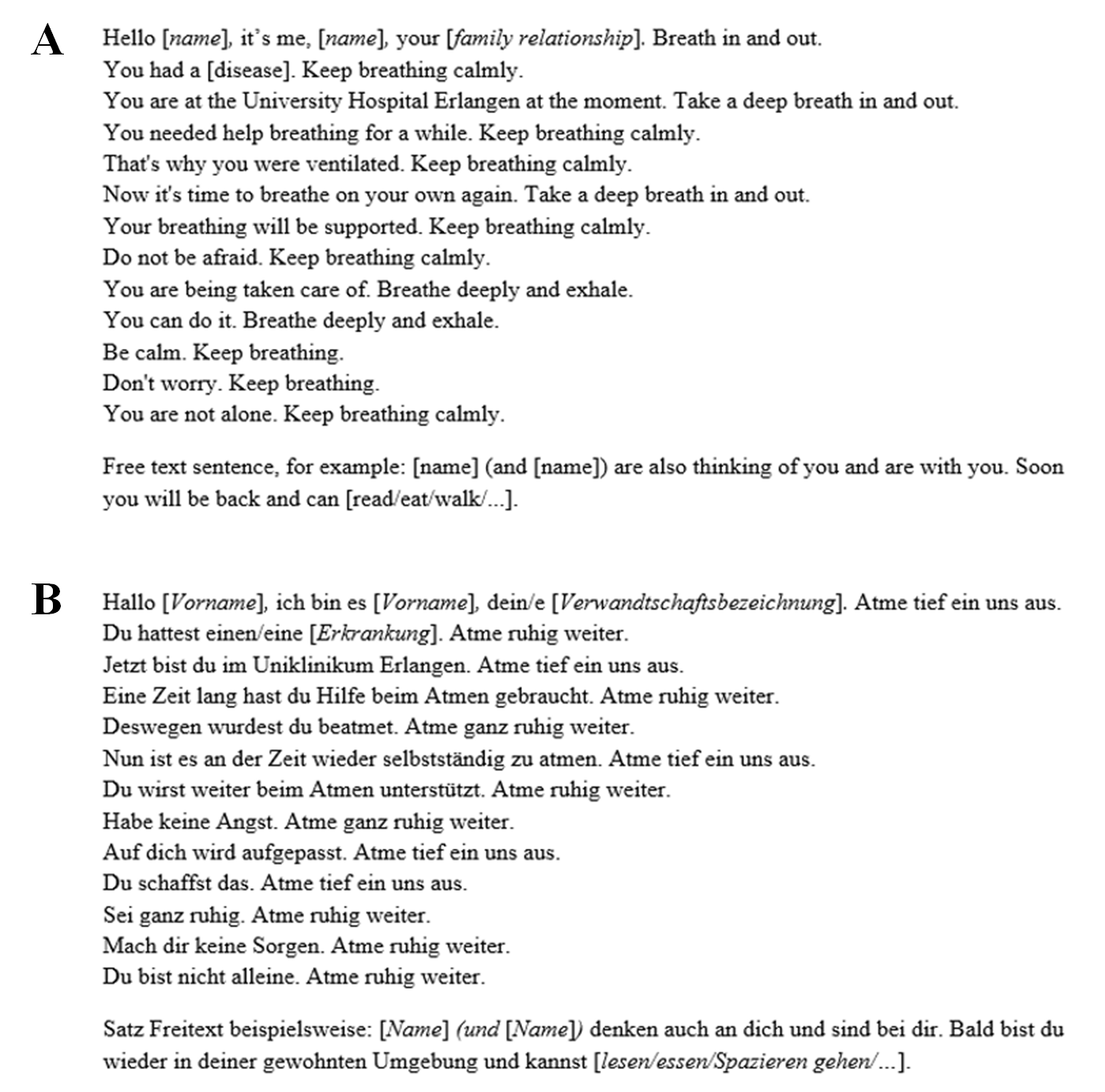


A predefined text - A) in English, B) in German - including information on the patient's condition and recurrent request to breath in and out was recorded as an audio file by one of the patient's relatives.

**Supplemental Figure 3: Illustration of study procedure and weaning process (simplified)**


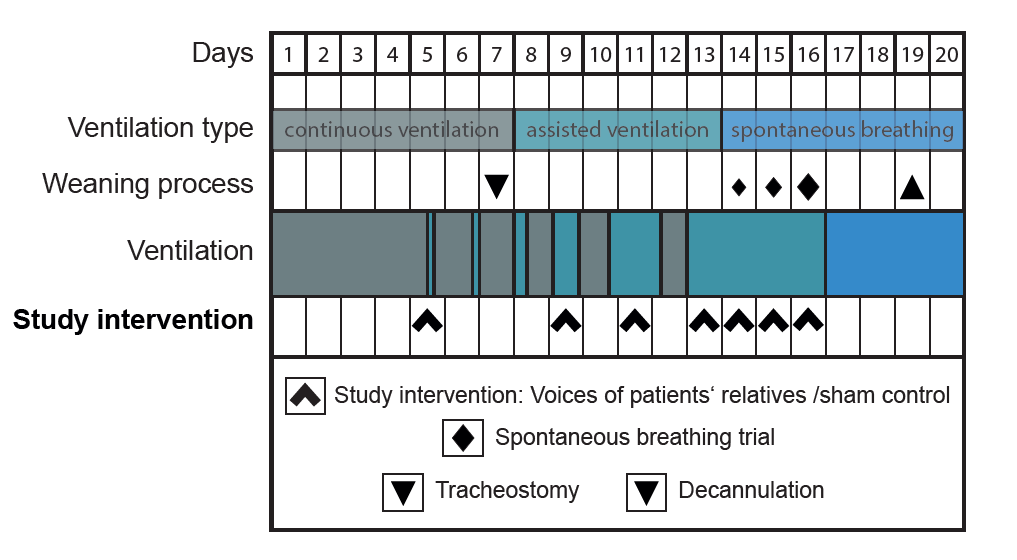


Audio recordings were administered when the first conversion from controlled to assisted mechanical ventilation was performed according to standard treatment. Thereafter, audio recordings were administered three times per day (in the morning between 6:00 and 8:00; at midday between 12:00 and 14:00 and in the evening between 18:00 and 20:00) in VOICE-Weaning II, but only during assisted mechanical ventilation. Spontaneous breathing trials (SBT), when intended according to standard treatment, were performed during administration of the audio recordings.

**Supplemental Table 4: Criteria for weaning not to be intended - according to Boles et al. (Eur Respir J. 2007) and adapted to neurological patients**

| **Clinical assessment** | |
| --- | --- |
| - Inadequate cough | |
| - Excessive tracheobronchial secretion | |
| - Disease acute phase for which the patient was intubated | |
| **Objective measurement** | |
| - Unstable clinical condition | |
|  | - Cardiovascular status (fC>140/min, systolic BP <90mmHg or >160mmHg, more than minimal vasopressors) |
|  | - Metabolic status (e.g. inacceptable electrolytes) |
| - Inadequate oxygenation | |
|  | - SaO2 ≤90% on FiO2 >40% or PaO2/FiO2 <150mmHg or PaO2/FiO2 <120mmHg in case of chronic hypoxemia (Horowitz index) |
|  | - PEEP>8cmH2O |
| - Inadequate pulmonary function | |
|  | - fR >35/min |
|  | - MIP>(-20 -) -25cmH2O |
|  | - VT ≤5ml/kg |
|  | - VC ≤10ml/kg |
|  | - fR/VT ≥105 breaths/min x l (RSBI) |
|  | - significant respiratory acidosis (pH≤7.25) |
| **Neurological condition** | |
| - Unstable neurological condition | |
|  | - sedation or inadequate mentation on sedation |
|  | - present or anticipated intracranial mass effect (e.g. midline shift >10mm or progression, basal cistern effacement or oculomotor disturbance, signs of transfalxial/transtentorial/transforaminal herniation) |
|  | - elevation of intracranial pressure (>20 cmH2O) and/or obstructive hydrocephalus |
|  | - severe vasospasms (>6 kHz in transcranial Doppler or determined by CT-A/CT-P) |
|  | - status epilepticus (determined by EEG) |
|  | - acute intracranial infection (without established and/or treated origin) |

Abbreviations: fC, cardiac frequency; BP, blood pressure; SaO2, arterial oxygen saturation; FiO2, inspiratory oxygen fraction; PaO2, arterial oxygen tension; PEEP, positive end-expiratory pressure; fR, respiratory frequency; MIP, maximal inspiratory pressure; VT, tidal volume; VC, vital capacity; RSBI, Rapid Shallow Breathing Index; CT, computed tomography; EEG, Electroencephalography.

**Supplemental Table 5: Criteria for failed spontaneous breathing trial (SBT) - according to Boles et al. (Eur Respir J. 2007) and MacIntyre et al. (Chest 2001)**

| **Objective measurements** |
| --- |
| - PaO2 ≤50(-60)mmHg on FiO2 ≥50% or SaO2<(85-)90% |
| - PaCO2 >50mmHg or an increase in PaCO2 >8mmHg |
| - pH <7,32 or a decrease in pH ≥0.07 pH units |
| - fR/VT >105 breaths/min x l |
| - fR>35 breaths/min or increased by ≥50% |
| - fC>140beats/min or increased by ≥20% |
| - systolic BP>180mmHg or increased by ≥20% |
| - systolic BP<90mmHg |
| - Cardiac arrhythmias |
| **Clinical assessment** |
| - Agitation and anxiety |
| - Depressed mental status |
| - Diaphoresis |
| - Cyanosis |
| - Evidence of increasing effort (increased accessory muscle activity, facial signs of distress, dyspnoea) |

Abbreviations: PaO2, arterial oxygen tension; FiO2, inspiratory oxygen fraction; SaO2, arterial oxygen saturation; PaCO2, arterial carbon dioxide tension; fR, respiratory frequency; VT, tidal volume; fC, cardiac frequency; BP, blood pressure.

**Supplemental Table 6: Weaning failure in the VOICE-WEANING trial**

| **Type of Weaning failure** | **ARPR group (n=11/21 [52.4%])** | **Control group**  **(n=14/22 [63.6%])** |
| --- | --- | --- |
| Reintubation and/or resumption of ventilatory support ≤48h following extubation | 0 (0.0%) | 1 (7.1%) |
| Death ≤48 hours following extubation | 1 (9.1%) | 2 (14.3%) |
| Failed spontaneous breathing trial | 10 (90.9%) | 11 (78.6%) |

Abbreviation: ARPR, audio recordings of patients' relatives.

**Supplemental Figure 4: Subgroup analyses**


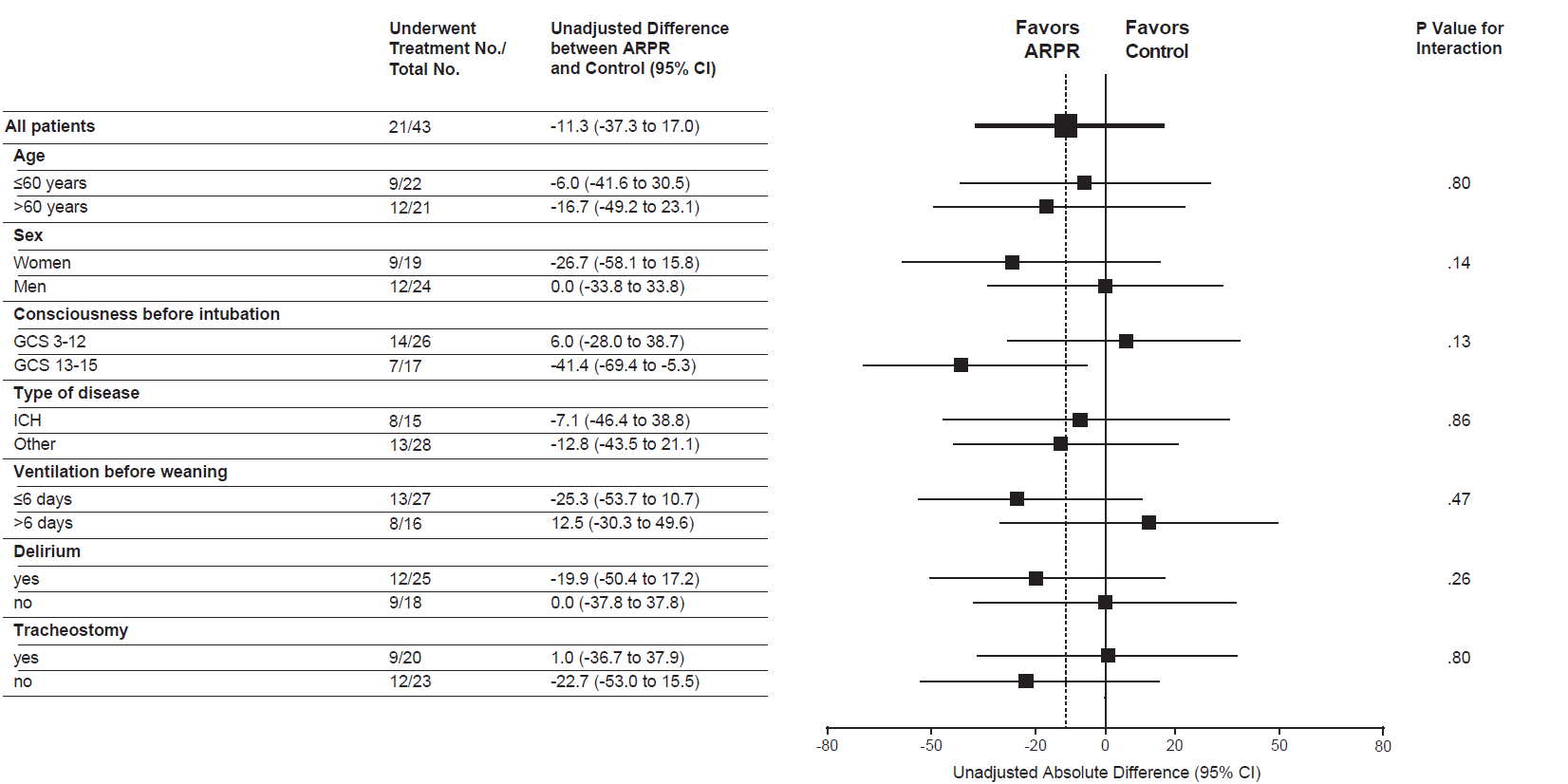


The forest plot shows unadjusted subgroup analyses for weaning failure using univariable logistic regression analysis to calculate P values for interaction. There was no evidence of heterogeneity of treatment effect among patient subgroups. Abbreviations: GCS, Glasgow Coma Scale; ICH, intracerebral hemorrhage; ARPR, audio recordings of patients’ relatives; CI, confidence interval. Subgroups were prespecified, but slightly modified due to limited patient numbers among the subgroups of consciousness before intubation (prespecified: 3-4; 5-12; 13-15) and type of disease (prespecified: intracerebral hemorrhage; ischemic stroke; subarachnoid hemorrhage; other diseases).

**Supplemental Figure 5: Exploratory subgroup analyses**


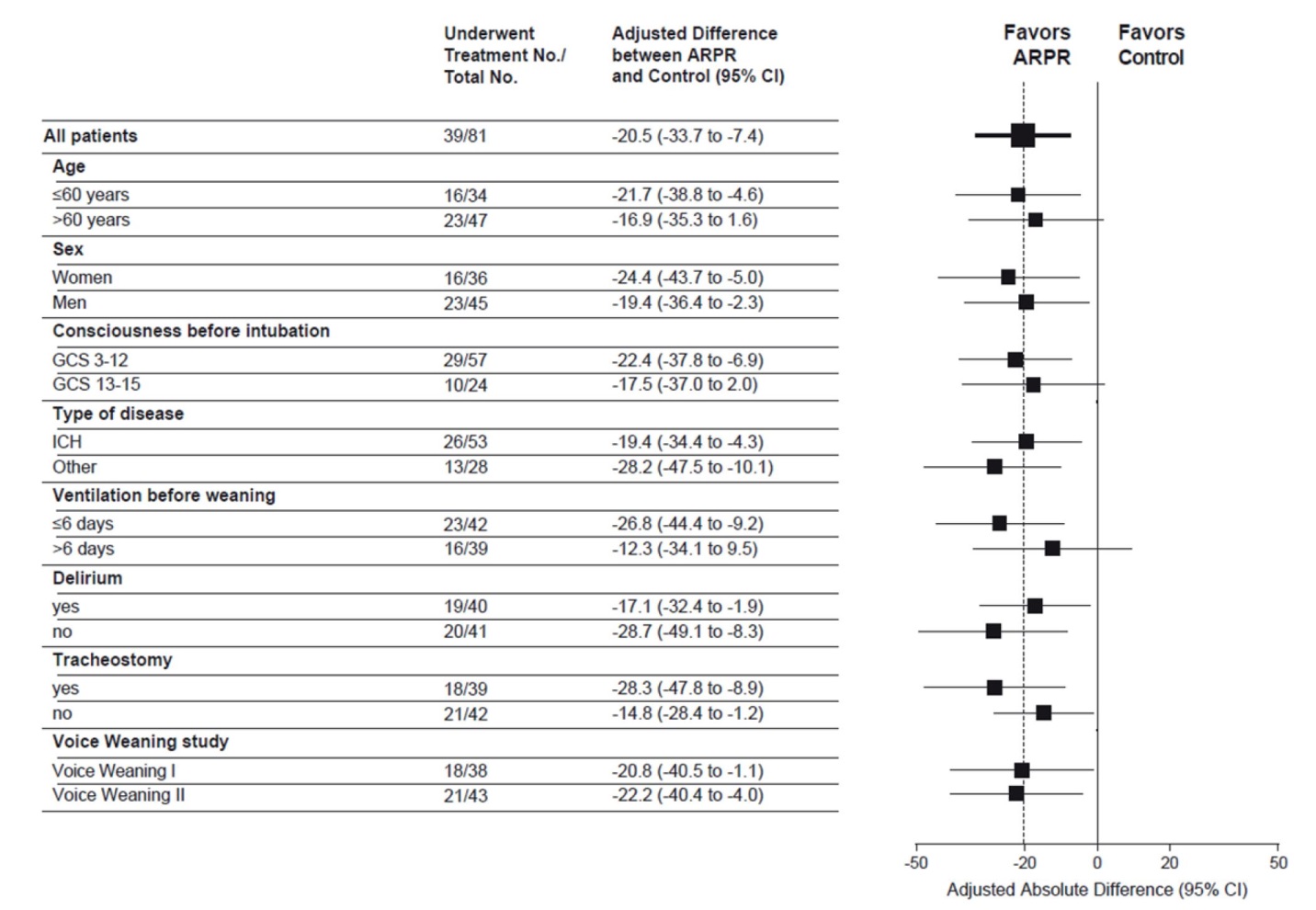


Exploratory subgroup analyses for the secondary outcome, duration of controlled mechanical ventilation after weaning initiation, were conducted using augmented inverse propensity weighted estimators to combine advantages of regression-adjustment and inverse-probability-weighted methods among pooled individual patient data of VOICE-WEANING I and VOICE-WEANING II studies. The first column represents patient subgroups (e.g. according to patient age ≤60 years versus >60 years). The second column represents the sample size for each subgroup (e.g. 16 patients in the ARPR group and 18 patients in the control group among patients ≤60 years of age). The third column represents adjusted differences between ARPR and control group in the duration of controlled mechanical ventilation in hours, i.e. average treatment effects. ARPR treatment favored the intervention group in the overall group (adjusted difference -20.5 hours; 95% confidence interval, -33.7 to -7.4; P<0.01) and across all subgroups, but limited patient numbers do not allow definite conclusions. Logistic regression models (augmented inverse probability weighting) were adjusted for age, gender, tobacco use, PaO2/FiO2 prior to weaning initiation, admission diagnosis (confounders identified in inter-group comparison) and additionally body mass index and GCS before intubation (outcome-specific confounders). Subgroups of the exploratory analysis were derived from prespecified subgroups of the primary outcome analysis (subgroups of consciousness before intubation [prespecified: 3-4; 5-12; 13-15] and type of disease [prespecified: intracerebral hemorrhage; ischemic stroke; subarachnoid hemorrhage; other diseases] were slightly modified due to limited patient numbers; Supplemental Fig. 4) except for Voice Weaning study parts. Abbreviations: ARPR, audio recordings of patients' relatives; GCS, Glasgow Coma Scale; ICH, intracerebral hemorrhage.

**Supplemental Figure 6: Temporal and regional patterns of alpha-delta ratio in response to treatment**


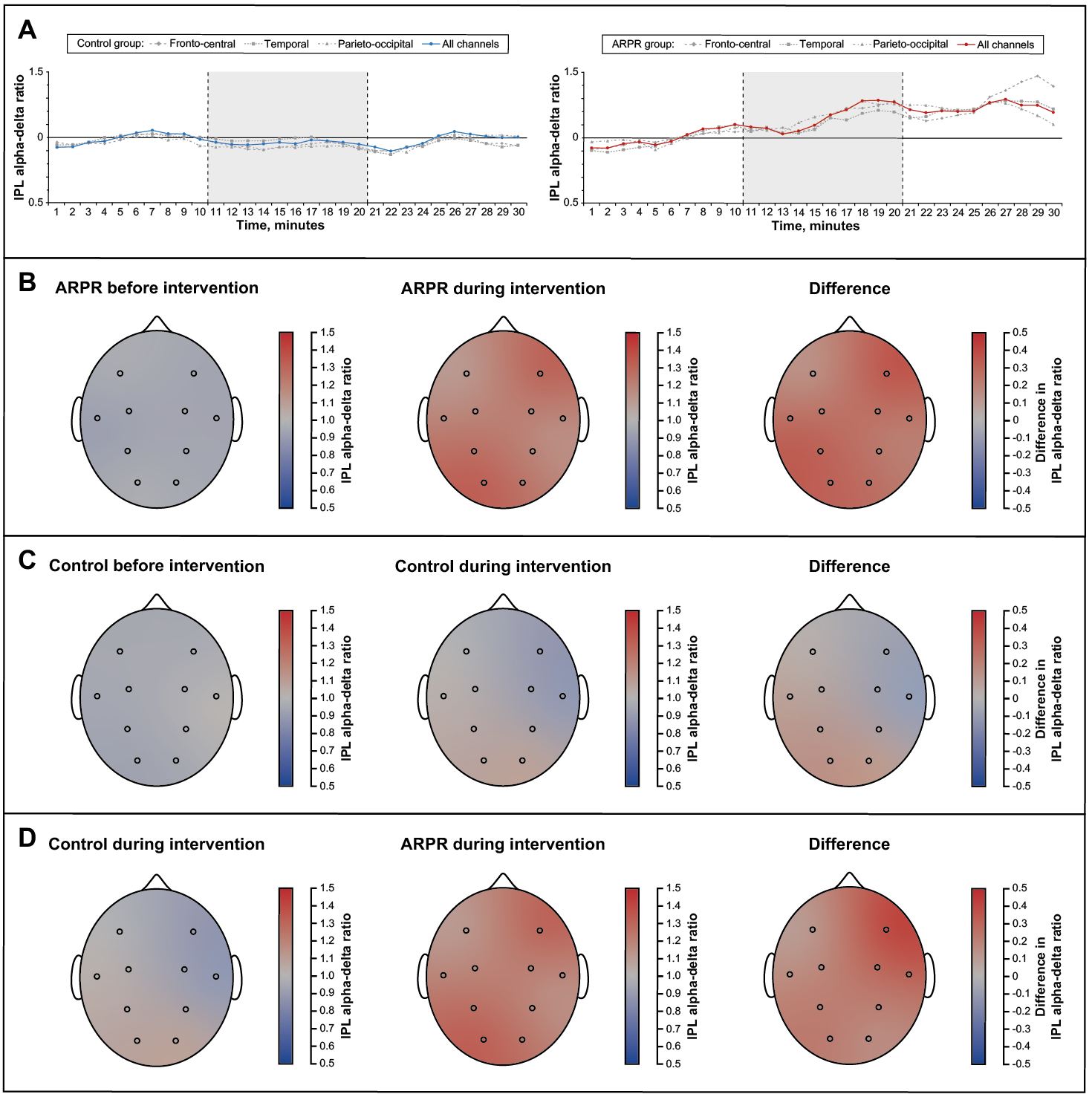


Exploratory EEG analyses were performed to assess alpha-delta ratio in response to ARPR. A) Temporal patterns of relative IPL alpha-delta ratio values: Before, during (gray area) and after treatment intervention for the control group (left, N=12) and the ARPR group (right, N=11). B) Regional patterns of relative IPL alpha-delta ratio values in the ARPR group before treatment intervention (left, N=11), during intervention (middle, N=11) and for the difference between during and before intervention (right, N=11; numbers represent mean differences, F8-C4: 0.35; 95% CI, 0.12 to 0.58; P<0.01; t=3.42 using paired samples t-test). C) Regional patterns of relative IPL alpha-delta ratio values in the control group before treatment intervention (left, N=12), during intervention (middle, N=12) and for the difference between during and before intervention (right, N=12; numbers represent mean differences, P3-O1: 0.14; 95% CI, -0.18 to 0.45; P=0.37; t=0.94 using paired samples t-test). D) Regional patterns of relative IPL alpha-delta ratio values in the control group during treatment intervention (left, N=12), in the ARPR group during intervention (middle, N=11) and for the difference between ARPR and control group during intervention (right, N=23; numbers represent mean differences, F8-C4: 0.40; 95% CI, 0.15 to 0.66; P<0.01; t=3.31 using independent samples t-test). Abbreviations: ARPR, audio recordings of patients' relatives; C3 and C4, central scalp electrode in position 3 and 4, respectively, according to the 10-20 system; EEG, electroencephalogram; F7 and F8, frontal scalp electrode in position 7 and 8, respectively; IPL, individual participant level; O1 and O2, occipital scalp electrode in position 1 and 2, respectively; P3 and P4, parietal scalp electrode in position 3 and 4, respectively; T3 and T4; temporal electrode in position 3 and 4, respectively.

**Clinical Trial Protocol**

**Voices of Patients' Relatives to Support Weaning**

**From Mechanical Ventilation in Patients**

**With Severe Brain Injury –**

**VOICE-WEANING, a Randomized Clinical Trial**

Short title: Voices of patients’ relatives to support weaning from mechanical ventilation (VOICE-WEANING)

Study Type: Interventional (clinical trial)

Study Registration: ClinicalTrials.gov Identifier: NCT03795623

Study Identifier: VOICE-WEANING

Study Sponsor: Friedrich-Alexander-Universität Erlangen-Nürnberg

Principal Investigator: Prof. Dr. med. Dr. Hagen Huttner

Department of Neurology

University Hospital Erlangen

Schwabachanlage 6

91054 Erlangen

Tal: +49 9131 8533001

e-mail: [hagen.huttner@uk-erlangen.de](mailto:hagen.huttner@uk-erlangen.de)

Funding Source: Johannes und Frieda Marohn-Stiftung

Protocol Version and Date: V2.0 - 29.10.2018

**SIGNATURE PAGE**

The VOICE-WEANING Trial, Final version 2.0, dated 29 October 2018

Written and approved by:


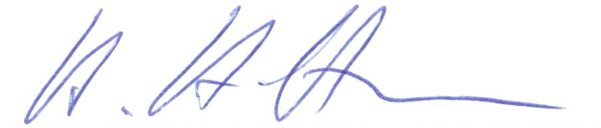


29 / October / 2018

__________________________________ __________________________________

Prof. Dr. med. Hagen B. Huttner Date

Chief Investigator

**TABLE OF CONTENTS**

[**PROTOCOL SYNOPSIS** 24](#_Toc161045064)

[**STUDY SUMMARY IN LOCAL LANGUAGE** 27](#_Toc161045065)

[**ABBREVIATIONS** 28](#_Toc161045066)

[**STUDY SCHEDULE** 30](#_Toc161045067)

[**1.** **STUDY ADMINISTRATIVE STRUCTURE** 32](#_Toc161045068)

[**1.1.** **Sponsor** 32](#_Toc161045069)

[**1.2.** **Principal Investigator(s)** 32](#_Toc161045070)

[**1.3.** **Laboratory** 33](#_Toc161045071)

[**1.4.** **Data Safety Monitoring Board** 33](#_Toc161045072)

[**2.** **ETHICAL AND REGULATORY ASPECTS** 33](#_Toc161045073)

[**2.1.** **Trial registration** 33](#_Toc161045074)

[**2.2.** **Ethics Committee and Regulatory Approvals** 34](#_Toc161045075)

[**2.3.** **Ethical conduct of the trial** 34](#_Toc161045076)

[**2.4.** **Patient Information and Informed Consent** 34](#_Toc161045077)

[**2.5.** **Participant privacy and confidentiality** 34](#_Toc161045078)

[**2.6.** **Early termination of the trial** 34](#_Toc161045079)

[**3.** **BACKGROUND AND RATIONALE** 35](#_Toc161045080)

[**3.1.** **Background** 35](#_Toc161045081)

[**3.2.** **Rationale for the Study** 35](#_Toc161045082)

[**3.3.** **Treatment Intervention** 36](#_Toc161045083)

[**3.4.** **Risks and benefits** 38](#_Toc161045084)

[**4.** **STUDY DESIGN** 38](#_Toc161045085)

[**4.1.** **General study design** 38](#_Toc161045086)

[**4.2.** **Randomization and Blinding** 38](#_Toc161045087)

[**4.3.** **Primary Outcome** 39](#_Toc161045088)

[**4.4.** **Secondary Outcomes** 40](#_Toc161045089)

[**5.** **STUDY POPULATION** 40](#_Toc161045090)

[**5.1.** **Recruitment** 40](#_Toc161045091)

[**5.2.** **Inclusion criteria** 40](#_Toc161045092)

[**5.3.** **Exclusion criteria** 40](#_Toc161045093)

[**6.** **STUDY ASSESSMENTS** 41](#_Toc161045094)

[**6.1.** **Study Schedule** 41](#_Toc161045095)

[**6.2.** **Study flow chart** 43](#_Toc161045096)

[**6.3.** **Assessment of outcomes** 43](#_Toc161045097)

[**6.3.1.** **Assessment of primary outcome** 43](#_Toc161045098)

[**6.3.2.** **Assessment of secondary outcomes** 43](#_Toc161045099)

[**6.4.** **Procedures at each visit** 44](#_Toc161045100)

[**6.4.1.** **Screening and enrolment period (day 1)** 44](#_Toc161045101)

[**6.4.2.** **Intervention period (day 2 to day 28)** 44](#_Toc161045102)

[**6.4.3.** **Follow-up (day 90)** 44](#_Toc161045103)

[**7.** **STATISTICAL METHODS** 45](#_Toc161045104)

[**7.1.** **Hypothesis** 45](#_Toc161045105)

[**7.2.** **Estimation of Sample Size** 45](#_Toc161045106)

[**7.3.** **Statistical methods** 45](#_Toc161045107)

[**7.4.** **Analysis population** 45](#_Toc161045108)

[**7.5.** **Missing data** 46](#_Toc161045109)

[**8.** **QUALITY ASSURANCE AND AUDIT** 46](#_Toc161045110)

[**8.1.** **Insurance** 46](#_Toc161045111)

[**8.2.** **Study Data** 46](#_Toc161045112)

[**8.3.** **Record Retention** 46](#_Toc161045113)

[**8.4.** **Monitoring and quality assurance of study procedures** 46](#_Toc161045114)

[**8.5.** **Audits and Inspections** 47](#_Toc161045115)

[**8.6.** **Confidentiality** 47](#_Toc161045116)

[**9.** **PUBLICATION AND DISSEMINATION POLICY** 47](#_Toc161045117)

[**10.** **FUNDING** 47](#_Toc161045118)

[**10.1.** **Funding source** 47](#_Toc161045119)

[**10.2.** **Participants payments** 48](#_Toc161045120)

[**11.** **REFERENCES** 49](#_Toc161045121)

[**12. APPENDICES** 51](#_Toc161045122)

# **PROTOCOL SYNOPSIS**

| **Title** | Voices of Patients' Relatives to Support Weaning From Mechanical Ventilation in Patients With Severe Brain Injury – VOICE-WEANING, a Randomized Clinical Trial |
| --- | --- |
| **Acronym** | VOICE-WEANING |
| **Short title** | Voices of patients’ relatives to support weaning from mechanical ventilation (VOICE-WEANING) |
| **Protocol Version** | V2.0, 29.10.2018 |
| **Trial registration** | clinicaltrials.gov (NCT03795623) |
| **Chief Investigator** | Prof. Dr. med. Hagen B. Huttner |
| **Study design** | Interventional Study Design |
| **Study Phase** | Phase 2 |
| **Background and Rationale** | Patients with severe brain injuries, such as ischemic stroke and intracranial hemorrhage, frequently require mechanical ventilation. Weaning of stroke patients is complicated by impaired consciousness and respiratory drive. Higher rates of weaning failure and delayed extubation (≥ 48h) lead to ventilator associated pneumonia, higher mortality and necessity of tracheostomy.  Therefore, improving the weaning of stroke patients from mechanical ventilation is warranted to prevent ventilator-associated complications and eventually improve clinical outcomes. |
| **Objective** | This single-center, randomized, clinical trial aims at demonstrating that voices of patients’ relatives support weaning from mechanical ventilation and reduce weaning failure in brain-injured patients. |
| **Outcomes** | Primary outcome:  The primary outcome is weaning failure defined as Failed spontaneous breathing trial, reintubation and/or resumption of ventilatory support ≤ 48h or death ≤48h following extubation  Secondary outcomes:  Secondary outcomes are: Duration of controlled ventilation, rate of tracheotomy, all-cause mortality rate at 90 days, rate of ICU delirium. |
| **Inclusion / Exclusion criteria** | The main inclusion criteria are intubation and controlled mechanical ventilation ≥48h due to a neurological disease; weaning from mechanical ventilation intended by the attending physician; obtained informed consent from the legal representative.  Main exclusion criteria are age < 18 years; history of psychiatric disease; weaning from mechanical ventilation not intended or decision to limit therapeutic interventions. |
| **Measurements and procedures** | Adult ICU-patients with controlled mechanical ventilation ≥48h due to a neurological disease will be included in the intensive care unit. A predefined text - including information on the patient’s condition and recurrent request to breath in and out - will be recorded as an audio file by one of the patient’s relatives. Patients will be randomly assigned in a 1:1 ratio to the conventional treatment arm or the Voice-Weaning arm. In the conventional arm, audio recordings will be muted by an independent person resulting in a mute recording without audio signals.  When conversion from controlled to assisted mechanical ventilation is intended according to standard treatment, audio recordings will be administered in repeat mode for 10 minutes and performed three times per day. If spontaneous breathing trials (SBT) are intended according to standard treatment, the audio recordings will be played during the SBT three times per day. |
| **Intervention** | Audio recordings of the patients relatives including information on the patient's condition and recurrent request to breath in and out. |
| **Number of Participants** | The sample size is computed with 80% power and a 5% α-risk for the hypothesis of Voice Weaning achieving a 15% absolute weaning failure reduction. The sample size is increased by 10% to correct for dropouts and lost to follow up: a maximum of 354 patients will be included and an interim analysis be performed after inclusion of 50% of the calculated subjects. |
| **Trial Duration** | 3 years |
| **Trial Schedule** | February 2019 – December 2021 |
| **Investigator** | Prof. Dr. med. Dr. Hagen Huttner  Stellv. Klinikdirektor Neurologie, Universität Erlangen-Nürnberg  University Hospital Erlangen  Schwabachanlage 6  91054 Erlangen  Tel.: +49 9131 85 44523  E-Mail: hagen.huttner@uk-erlangen.de |
| **GCP Statement** | This study will be conducted in compliance with the protocol, the current version of the Declaration of Helsinki, the ICH-GCP as well as all national legal and regulatory requirements. |

# **STUDY SUMMARY IN LOCAL LANGUAGE**

Das Weaning d.h. das Entwöhnen vom Beatmungsgerät erweist sich bei neurologischen Intensivpatienten aufgrund von zerebral bedingten Atemantriebsstörungen schwieriger als in anderen Fachdisziplinen. Gerade die erste Umstellung von einem kontrollierten in einen assistierten Beatmungsmodus sowie im weiteren Verlauf die Initiierung von intermittierenden Spontanatemversuchen - als Voraussetzungen für eine erfolgreiche Beendigung der maschinellen Beatmung - stellen meist eine langwierige Herausforderung dar. Vorteile der assistierten Beatmung sind eine physiologische Atemmotorik mit besserer Zwerchfellmotilität und Säure-Basen-Haushalt-Regulation, weniger Stress und Schmerz somit erhöhter Patientenkomfort und daraus resultierend ein geringerer Bedarf an analgosedierenden Medikamenten. Entsprechend gilt die Umstellung in die assistierte Beatmung als eines der ersten Ziele im Weaning-Prozess, welches möglichst frühzeitig und dauerhaft erreicht werden sollte. Jedoch haben die Patienten in dieser kritischen Umstellungsphase häufig eine sogenannte Kommandoatmung und lassen sich nur durch Aufforderungen zum selbstständigen Atmen am Gerät anweisen; d.h. sobald keine Stimulation mehr erfolgt, fällt der Patient häufig zurück in die sog. Apnoeventilation und damit wieder in den kontrollierten Beatmungsmodus. Des Weiteren stellen Spontanatemversuche prognostisch wichtige Bausteine zur Beurteilung des Entwöhnens vom Respirator dar, auch an dieser Stelle sind Atemkommandos wichtig, um solche Versuche zu unterstützen, um Stress für den Patienten zu reduzieren, und die Chancen auf eine Extubation zu erhöhen. Allerdings ist grundsätzlich anzunehmen, aufgrund der heutigen personellen und organisatorischen Strukturen auf Intensivstationen, dass eine solch individuelle Betreuung der Patienten nicht immer bzw. nur selten konsequent gewährleistet werden kann.

Ziel dieser Studie ist es daher zu untersuchen, ob aufgezeichnete Angehörigen-Stimmen effektiv und sicher den Weaning-Prozess von neurologischen Patienten unterstützen können.

# **ABBREVIATIONS**

ARPR Audio recordings of patients’ relatives

BP Blood pressure

CAM-ICU Confusion Assessment Method for intensive care unit

CRF Case Report Form

DSMB Data Safety Monitoring Board

EC Ethics committee

fC Cardiac frequency

fR Respiratory frequency

FiO2 Inspiratory oxygen fraction

GCP Good Clinical Practice

GCS Glasgow Coma Scale

IC Informed consent

ICH Intracerebral hemorrhage

ICU Intensive Care Unit

MIP Maximal inspiratory pressure;

mRS Modified Rankin Scale

NA Not applicable

NIHSS National Institute of Health Score Scale

PaCO2 Arterial carbon dioxide tension

PaO2 Arterial oxygen tension

PEEP Positive end-expiratory pressure

PI Principal Investigator

RASS Richmond Agitation-Sedation Scale

REC Research Ethics Committee

SAH Subarachnoid hemorrhage

SaO2 Arterial oxygen saturation

SBT Spontaneous breathing trial

VC Vital capacity

VT Tidal volume

# **STUDY SCHEDULE**

| **Study period** | **Screening**  **/enrolment** | **Intervention period** | | | | | | | | | | | | | | | | | | | | | | | | | | | **Follow-up** |
| --- | --- | --- | --- | --- | --- | --- | --- | --- | --- | --- | --- | --- | --- | --- | --- | --- | --- | --- | --- | --- | --- | --- | --- | --- | --- | --- | --- | --- | --- |
| **Days** | **1** | **2** | **3** | **4** | **5** | **6** | **7** | **8** | **9** | **10** | **11** | **12** | **13** | **14** | **15** | **16** | **17** | **18** | **19** | **20** | **21** | **22** | **23** | **24** | **25** | **26** | **27** | **28** | **90** |
| Visit Window | None | ±4h | ±4h | ±4h | ±4h | ±4h | ±4h | ±4h | ±4h | ±4h | ±4h | ±4h | ±4h | ±4h | ±4h | ±4h | ±4h | ±4h | ±4h | ±4h | ±4h | ±4h | ±4h | ±4h | ±4h | ±4h | ±4h | ±4h | ±2d |
| **Assessments** |  |  |  |  |  |  |  |  |  |  |  |  |  |  |  |  |  |  |  |  |  |  |  |  |  |  |  |  |  |
| Incl/excl criteria | X |  |  |  |  |  |  |  |  |  |  |  |  |  |  |  |  |  |  |  |  |  |  |  |  |  |  |  |  |
| Informed consent | X |  |  |  |  |  |  |  |  |  |  |  |  |  |  |  |  |  |  |  |  |  |  |  |  |  |  |  |  |
| Medical history | X |  |  |  |  |  |  |  |  |  |  |  |  |  |  |  |  |  |  |  |  |  |  |  |  |  |  |  |  |
| Admission details | X |  |  |  |  |  |  |  |  |  |  |  |  |  |  |  |  |  |  |  |  |  |  |  |  |  |  |  |  |
| Randomization | X |  |  |  |  |  |  |  |  |  |  |  |  |  |  |  |  |  |  |  |  |  |  |  |  |  |  |  |  |
| NIHSS | X | X | X | X | X | X | X | X | X | X | X | X | X | X | X | X | X | X | X | X | X | X | X | X | X | X | X | X |  |
| GCS | X | X | X | X | X | X | X | X | X | X | X | X | X | X | X | X | X | X | X | X | X | X | X | X | X | X | X | X |  |
| CAM-ICU |  | X | X | X | X | X | X | X | X | X | X | X | X | X | X | X | X | X | X | X | X | X | X | X | X | X | X | X |  |
| Physical examination | X | X | X | X | X | X | X | X | X | X | X | X | X | X | X | X | X | X | X | X | X | X | X | X | X | X | X | X |  |
| Vital signs | X | X | X | X | X | X | X | X | X | X | X | X | X | X | X | X | X | X | X | X | X | X | X | X | X | X | X | X |  |
| Ventilation parameters | X | X | X | X | X | X | X | X | X | X | X | X | X | X | X | X | X | X | X | X | X | X | X | X | X | X | X | X |  |
| Medication | X | X | X | X | X | X | X | X | X | X | X | X | X | X | X | X | X | X | X | X | X | X | X | X | X | X | X | X |  |
| In-hospital complications |  | X | X | X | X | X | X | X | X | X | X | X | X | X | X | X | X | X | X | X | X | X | X | X | X | X | X | X |  |
| Cognitive deficits, headache |  |  |  |  |  |  |  |  |  |  |  |  |  |  |  |  |  |  |  |  |  |  |  |  |  |  |  |  | X |
| Modified Ranking Scale | X |  |  |  |  |  |  |  |  |  |  |  |  |  |  |  |  |  |  |  |  |  |  |  |  |  |  |  | X |
| EQ-5D |  |  |  |  |  |  |  |  |  |  |  |  |  |  |  |  |  |  |  |  |  |  |  |  |  |  |  |  | X |

# **STUDY ADMINISTRATIVE STRUCTURE**

## **Sponsor**

This study is an investigator-initiated clinical trial.

The sponsor is the Friedrich-Alexander-Universität Erlangen-Nürnberg, represented by the Chief Investigator:

Prof. Dr. med. Dr. Hagen Huttner

Stellv. Klinikdirektor Neurologie, Universität Erlangen-Nürnberg

University Hospital Erlangen

Schwabachanlage 6

91054 Erlangen

Tel.: +49 9131 85 44523

E-Mail: hagen.huttner@uk-erlangen.de

## **Principal Investigator(s)**

PD Dr. med. Joji Kuramatsu

Neurologie, Universität Erlangen-Nürnberg

University Hospital Erlangen

Schwabachanlage 6

91054 Erlangen

Tel.: +49 9131 85 33001

E-Mail: joji.kuramatsu@uk-erlangen.de

Dr. med. Maximilian Sprügel

Neurologie, Universität Erlangen-Nürnberg

University Hospital Erlangen

Schwabachanlage 6

91054 Erlangen

Tel.: +49 9131 85 33001

E-Mail: maximilian.spruegel@uk-erlangen.de

## **Laboratory**

Not applicable as there are no study-specific laboratory analyses.

## **Data Safety Monitoring Board**

Members of the Data Safety Monitoring Board:

PD Dr. med. Stefan Gerner

Psychiatrie, Universität Erlangen-Nürnberg

University Hospital Erlangen

Schwabachanlage 6

91054 Erlangen

Tel.: +49 9131 85 33001

E-Mail: stefan.gerner@uk-erlangen.de

Dr. med. Julia Köhn

Neurologie, Universität Erlangen-Nürnberg

University Hospital Erlangen

Schwabachanlage 6

91054 Erlangen

Tel.: +49 9131 85 33001

E-Mail: julia.koehn@uk-erlangen.de

# **ETHICAL AND REGULATORY ASPECTS**

## **Trial registration**

The trial is registered at clinicaltrials.gov (NCT03795623).

## **Ethics Committee and Regulatory Approvals**

Study protocol, information and consent forms and other study-specific documents must be approved from a properly constituted Research Ethics Committee (REC) in agreement with local legal requirements, before the study will be initiated. Protocol amendments requiring REC approval will not be instituted until the amendment and revised documents have been approved.

## **Ethical conduct of the trial**

The study will be conducted in accordance to the protocol and with principles of the current version of the Declaration of Helsinki and the guidelines of Good Clinical Practice (GCP).

## **Patient Information and Informed Consent**

Eligible patients are unconscious and ventilated at the time of inclusion and not able to provide informed consent (IC) for study participation. Therefore, IC will be obtained from legal representatives of eligible patients following institutional and national REC policy. Post-hoc IC of included patients is required when patients regain capacity to give informed consent during the study period. If post-hoc IC is refused, patients will be withdrawn from the study and collected data will not be used for study purposes.

## **Participant privacy and confidentiality**

The principle of the participant's right to privacy and local privacy laws are uphold by the investigators. The minimum required information will be collected in the CRF, held securely and access to the information will be limited to study staff, investigators and relevant regulatory authorities.

## **Early termination of the trial**

The trial may be terminated prematurely by the Chief Investigator under certain circumstances, e.g.:

- Insufficient recruitment of participants
- Early evidence of benefit or harm of the study intervention

# **BACKGROUND AND RATIONALE**

## **Background**

Patients with severe brain injuries, such as ischemic stroke, intracranial hemorrhage, meningoencephalitis or status epilepticus, frequently require mechanical ventilation ([9-12](#_ENREF_9)). However, an altered level of consciousness and depressed respiratory drive complicate weaning from ventilation ([13](#_ENREF_13), [14](#_ENREF_14)). Notably the transition from controlled to assisted ventilation is challenging in disoriented patients with neurological deficits. Agitation and discomfort in brain-injured patients aggravates discoordination between the patient and the ventilator resulting in a patient “fighting the ventilator” ([15](#_ENREF_15), [16](#_ENREF_16)). This phenomenon causes discomfort, gas exchange deterioration and cardiovascular impairment, and frequently requires deeper sedation and prolonged controlled ventilation. As a result, higher rates of weaning failure and delayed extubation lead to ventilator associated pneumonia, necessity of tracheostomy and increased mortality ([10](#_ENREF_10), [12](#_ENREF_12), [16](#_ENREF_16), [17](#_ENREF_17)). Therefore, improving the weaning of brain-injured patients from mechanical ventilation is warranted to prevent ventilator-associated complications and eventually improve clinical outcomes ([17](#_ENREF_17)).

## **Rationale for the Study**

Intensive support by care providers including recurrent stimulation and request to breath in and out improves weaning of brain-injured patients and the transition from controlled to assisted ventilation, but limited resources undermine broad application in clinical routine ([18](#_ENREF_18), [19](#_ENREF_19)). Involving family members of critically ill patients into intensive care treatment improved patient-centered care and reduced the length of stay in the intensive care unit (ICU), but implementation in clinical routine is challenging ([20](#_ENREF_20)). However, the combination of intensive weaning support by patients’ relatives may improve weaning specifically in brain-injured patients.

## **Treatment Intervention**

A predefined text - including information on the patient's condition and recurrent request to breath in and out - will be recorded as an audio file by one of the patient's relatives at the neurological ICU using a digital recording device. Voice recordings will be transferred to a secure server. Audio editing will be performed by an independent person unblinded to randomization using an open-source audio software (www.audacityteam.org/) to reduce the dynamic range, to insert an initial audio notification (request to put the headphones on the patient’s head), and to create ARPR of approximately 10-minute length by repeating the voice recordings with audio breaks of 0.25 minutes in between. For the sham-control group, ARPR will be muted resulting in mute audio recordings without audio signals except for the initial audio notification. The independent person will transfer the audio recordings to a portable media player.

Predefined text in A) English and B) local language (German) for the audio recordings:


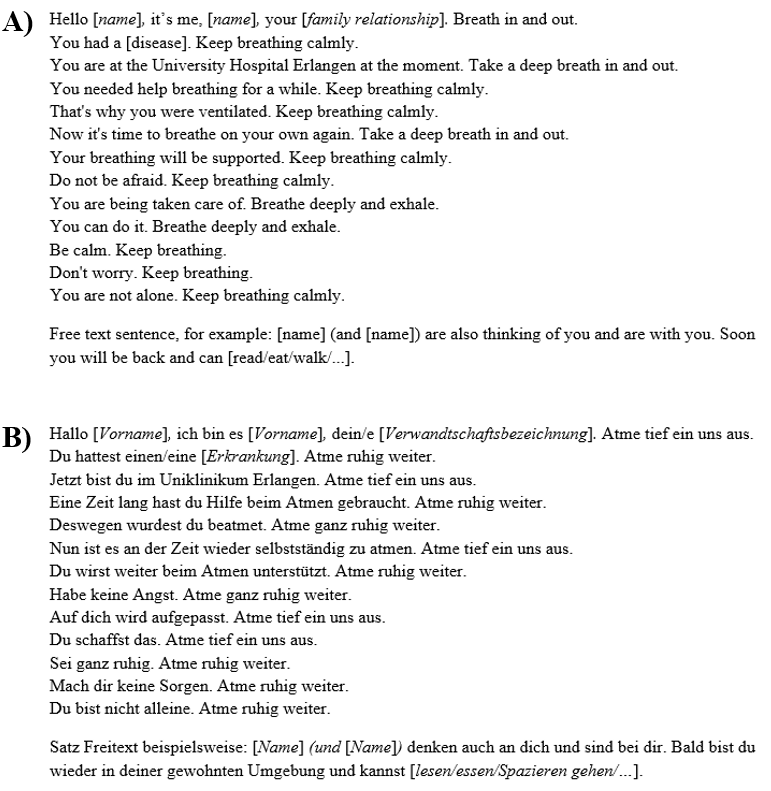


ARPR will be administered when the first conversion from controlled to assisted mechanical ventilation is performed according to standard treatment. Thereafter, audio recordings will be administered three times per day (in the morning between 6:00 and 8:00; at midday between 12:00 and 14:00 and in the evening between 18:00 and 20:00), but only during assisted mechanical ventilation. Spontaneous breathing trials (SBT), when intended according to standard treatment, will be performed during administration of the audio recordings.

Readiness to wean is assessed using criteria according to current guidelines adapted to neurological patients ([2](#_ENREF_2));

| **Clinical assessment** | |
| --- | --- |
| - Inadequate cough | |
| - Excessive tracheobronchial secretion | |
| - Disease acute phase for which the patient was intubated | |
| **Objective measurement** | |
| - Unstable clinical condition | |
|  | - Cardiovascular status (fC>140/min, systolic BP <90mmHg or >160mmHg, more than minimal vasopressors) |
|  | - Metabolic status (e.g. inacceptable electrolytes) |
| - Inadequate oxygenation | |
|  | - SaO2 ≤90% on FiO2 >40% or PaO2/FiO2 <150mmHg or PaO2/FiO2 <120mmHg in case of chronic hypoxemia (Horowitz index) |
|  | - PEEP>8cmH2O |
| - Inadequate pulmonary function | |
|  | - fR >35/min |
|  | - MIP>(-20 -) -25cmH2O |
|  | - VT ≤5ml/kg |
|  | - VC ≤10ml/kg |
|  | - fR/VT ≥105 breaths/min x l (RSBI) |
|  | - significant respiratory acidosis (pH≤7.25) |
| **Neurological condition** | |
| - Unstable neurological condition | |
|  | - sedation or inadequate mentation on sedation |
|  | - present or anticipated intracranial mass effect (e.g. midline shift >10mm or progression, basal cistern effacement or oculomotor disturbance, signs of transfalxial/transtentorial/transforaminal herniation) |
|  | - elevation of intracranial pressure (>20 cmH2O) and/or obstructive hydrocephalus |
|  | - severe vasospasms (>6 kHz in transcranial Doppler or determined by CT-A/CT-P) |
|  | - status epilepticus (determined by EEG) |
|  | - acute intracranial infection (without established and/or treated origin) |

## **Risks and benefits**

The study intervention may facilitate transition from controlled to assisted mode of ventilation thereby reducing the duration of ventilation and associated complications. Furthermore, including familiar voices of patients’ relatives could promote a feeling of comfort and security.

Auditory stimulations may increase the incidence of delirium. However, studies evaluating the effect of voices of patients’ relatives in neurological intensive care patients did not suggest such negative effects ([21](#_ENREF_21), [22](#_ENREF_22)).

# **STUDY DESIGN**

## **General study design**

VOICE-WEANING is a single-center, randomized (1:1) sham-controlled (care provider, investigator, outcomes assessor) study to assess the feasibility, safety and efficacy of audio recordings of patients' relatives (ARPR) to support weaning in patients with severe brain injury.

## **Randomization and Blinding**

Patients will be randomized with equal probabilities to the treatment group (ARPR) or the sham-control group (muted ARPR) using an online tool ([www.randomizer.at/](http://www.randomizer.at/)). Randomization is generated in permuted blocks and stratified for disease entity (ischemic stroke, intracerebral hemorrhage, subarachnoid hemorrhage and other diseases), age (≤60 years and >60years) and Glasgow Coma Scale (GCS) before intubation (3-4, 5-12, 13-15) ([3](#_ENREF_3), [4](#_ENREF_4)). Masking of patients is not possible owing to the intervention type. Care providers, investigators, outcome assessors and patients’ relatives are blinded to group assignment. The data safety monitoring board may have access to unblinded data but will have no contact with study participants.

## **Primary Outcome**

Primary outcome is the rate of weaning failure within 28 days after start of ventilation, or until 48 hours after extubation, or discharge from intensive care, whichever came first. Weaning failure is defined as reintubation and/or resumption of ventilatory support within 48 hours following extubation or death within 48 hours following extubation or failed SBT.

Criteria for SBT failure are defined according to current guidelines ([2](#_ENREF_2), [23](#_ENREF_23));

| **Objective measurements** |
| --- |
| - PaO2 ≤50(-60)mmHg on FiO2 ≥50% or SaO2<(85-)90% |
| - PaCO2 >50mmHg or an increase in PaCO2 >8mmHg |
| - pH <7,32 or a decrease in pH ≥0.07 pH units |
| - fR/VT >105 breaths/min x l |
| - fR>35 breaths/min or increased by ≥50% |
| - fC>140beats/min or increased by ≥20% |
| - systolic BP>180mmHg or increased by ≥20% |
| - systolic BP<90mmHg |
| - Cardiac arrhythmias |
| **Clinical assessment** |
| - Agitation and anxiety |
| - Depressed mental status |
| - Diaphoresis |
| - Cyanosis |
| - Evidence of increasing effort (increased accessory muscle activity, facial signs of distress, dyspnoea) |

## **Secondary Outcomes**

Secondary outcomes are duration of controlled mechanical ventilation; rate of tracheotomy within 28 days after start of ventilation or discharge from intensive care, whichever came first; rate of all-cause mortality at 90 days after start of ventilation; and rate of ICU delirium within 28 days after start of ventilation or discharge from intensive care, whichever came first. Delirium was defined according to the Confusion Assessment Method for intensive care unit (CAM-ICU) ([1](#_ENREF_1)).

# **STUDY POPULATION**

## **Recruitment**

Three hundred and fifty four (354) patients will be recruited at the Department of Neurology, University of Erlangen-Nürnberg: All patients at the neurological Intensive care unit, meeting inclusion criteria and not presenting any exclusion criteria, will be considered. There will be no reimbursement for trial participants.

## **Inclusion criteria**

- Intubation and controlled mechanical ventilation ≥48h due to a neurological disease
- Weaning from mechanical ventilation intended by the attending physician
- Obtained informed consent from the legal representative

## **Exclusion criteria**

- Age < 18 years
- History of psychiatric disease
- Weaning from mechanical ventilation not intended or decision to limit therapeutic interventions

# **STUDY ASSESSMENTS**

## **Study Schedule**

| **Study period** | **Screening**  **/enrolment** | **Intervention period** | | | | | | | | | | | | | | | | | | | | | | | | | | | **Follow-up** |
| --- | --- | --- | --- | --- | --- | --- | --- | --- | --- | --- | --- | --- | --- | --- | --- | --- | --- | --- | --- | --- | --- | --- | --- | --- | --- | --- | --- | --- | --- |
| **Days** | **1** | **2** | **3** | **4** | **5** | **6** | **7** | **8** | **9** | **10** | **11** | **12** | **13** | **14** | **15** | **16** | **17** | **18** | **19** | **20** | **21** | **22** | **23** | **24** | **25** | **26** | **27** | **28** | **90** |
| Visit Window | None | ±4h | ±4h | ±4h | ±4h | ±4h | ±4h | ±4h | ±4h | ±4h | ±4h | ±4h | ±4h | ±4h | ±4h | ±4h | ±4h | ±4h | ±4h | ±4h | ±4h | ±4h | ±4h | ±4h | ±4h | ±4h | ±4h | ±4h | ±2d |
| **Assessments** |  |  |  |  |  |  |  |  |  |  |  |  |  |  |  |  |  |  |  |  |  |  |  |  |  |  |  |  |  |
| Incl/excl criteria | X |  |  |  |  |  |  |  |  |  |  |  |  |  |  |  |  |  |  |  |  |  |  |  |  |  |  |  |  |
| Informed consent | X |  |  |  |  |  |  |  |  |  |  |  |  |  |  |  |  |  |  |  |  |  |  |  |  |  |  |  |  |
| Medical history | X |  |  |  |  |  |  |  |  |  |  |  |  |  |  |  |  |  |  |  |  |  |  |  |  |  |  |  |  |
| Admission details | X |  |  |  |  |  |  |  |  |  |  |  |  |  |  |  |  |  |  |  |  |  |  |  |  |  |  |  |  |
| Randomization | X |  |  |  |  |  |  |  |  |  |  |  |  |  |  |  |  |  |  |  |  |  |  |  |  |  |  |  |  |
| NIHSS | X | X | X | X | X | X | X | X | X | X | X | X | X | X | X | X | X | X | X | X | X | X | X | X | X | X | X | X |  |
| GCS | X | X | X | X | X | X | X | X | X | X | X | X | X | X | X | X | X | X | X | X | X | X | X | X | X | X | X | X |  |
| CAM-ICU |  | X | X | X | X | X | X | X | X | X | X | X | X | X | X | X | X | X | X | X | X | X | X | X | X | X | X | X |  |
| Physical examination | X | X | X | X | X | X | X | X | X | X | X | X | X | X | X | X | X | X | X | X | X | X | X | X | X | X | X | X |  |
| Vital signs | X | X | X | X | X | X | X | X | X | X | X | X | X | X | X | X | X | X | X | X | X | X | X | X | X | X | X | X |  |
| Ventilation parameters | X | X | X | X | X | X | X | X | X | X | X | X | X | X | X | X | X | X | X | X | X | X | X | X | X | X | X | X |  |
| Medication | X | X | X | X | X | X | X | X | X | X | X | X | X | X | X | X | X | X | X | X | X | X | X | X | X | X | X | X |  |
| In-hospital complications |  | X | X | X | X | X | X | X | X | X | X | X | X | X | X | X | X | X | X | X | X | X | X | X | X | X | X | X |  |
| Cognitive deficits, headache |  |  |  |  |  |  |  |  |  |  |  |  |  |  |  |  |  |  |  |  |  |  |  |  |  |  |  |  | X |
| Modified Ranking Scale | X |  |  |  |  |  |  |  |  |  |  |  |  |  |  |  |  |  |  |  |  |  |  |  |  |  |  |  | X |
| EQ-5D |  |  |  |  |  |  |  |  |  |  |  |  |  |  |  |  |  |  |  |  |  |  |  |  |  |  |  |  | X |

## **Study flow chart**


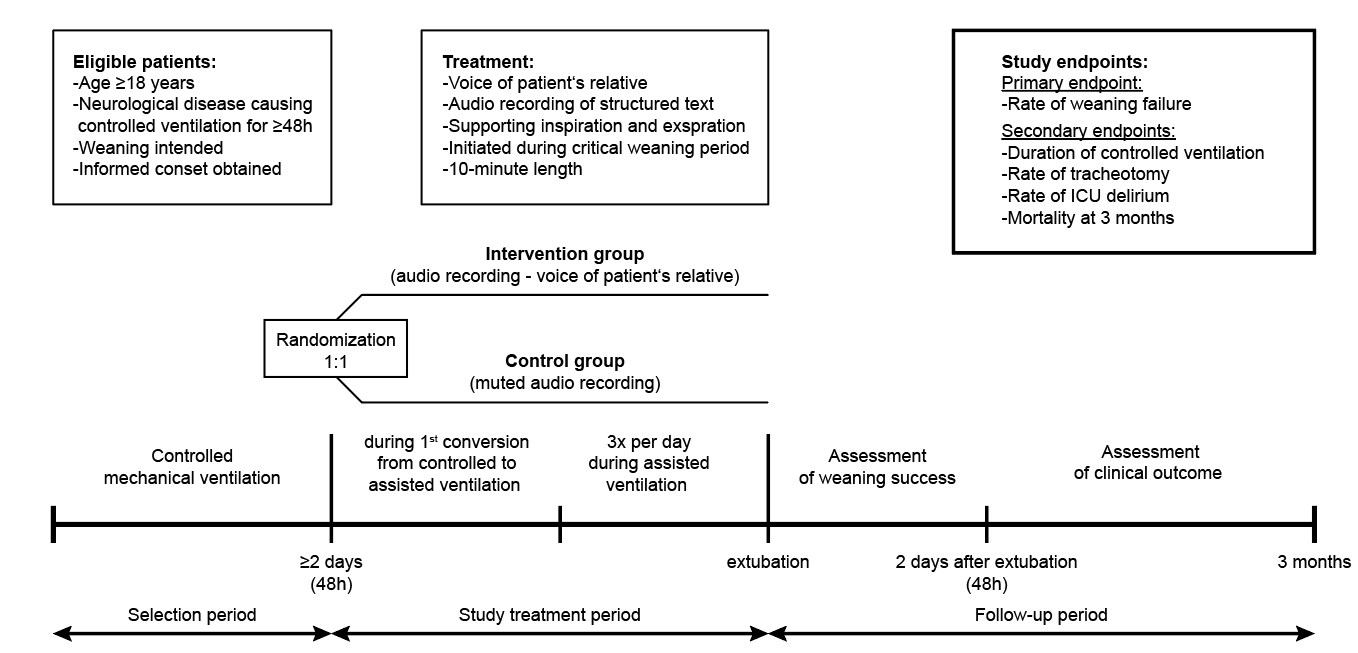


## **Assessment of outcomes**

### **Assessment of primary outcome**

Rate of weaning failure is assessed through daily visits at the ICU bedside at 10:00 am (±4hours) by a study team member blinded to the study intervention. The study visits are conducted within 28 days after start of ventilation, or until 48 hours after extubation, or discharge from intensive care, whichever came first.

### **Assessment of secondary outcomes**

The following secondary outcomes are assessed through daily visits at the ICU bedside at 10:00 am (±4hours) by a study team member blinded to the study intervention; duration of controlled mechanical ventilation, rate of tracheotomy and rate of ICU delirium. The study visits are conducted within 28 days after start of ventilation, or until 48 hours after extubation, or discharge from intensive care, whichever came first.

The secondary outcome rate of all-cause mortality at 90 days is assessed at 90 ± 2 days after randomization through a structured telephone interview by trained personnel (as described in section 6.4.3), who is not aware of the treatment allocation.

Other outcomes of interest are assessed through daily visits at the ICU bedside at 10:00 am (±4hours) by a study team member blinded to the study intervention; GCS, RASS, SBT, medication, eye movement, upper-airway functions and fluid balance.

## **Procedures at each visit**

### **Screening and enrolment period (day 1)**

Screening will take place at the neurological ICU to assess study inclusion and exclusion criteria. When informed consent from legal representatives is obtained for eligible patients, baseline assessment, audio recordings and randomization will be performed. Demographics, medical history, admission details, NIHSS, GCS, physical examination, vital signs, ventilation parameters, medication and premorbid mRS will be assessed. Further parameters will be collected from medical charts. Voices of patients’ relatives will be recorded using a predefined text as described in section 3.3.

### **Intervention period (day 2 to day 28)**

During this study period, the treatment intervention (ARPR) will be administered as described in section 3.3. NIHSS, GCS, CAM-ICU, physical examination, vital signs, ventilation parameters (including assessment of weaning success), medication and in-hospital complications will be assessed by daily study visits performed at the ICU bedside at 10:00 am (±4hours).

### **Follow-up (day 90)**

Clinical outcome will be assessed through a structured telephone interview (to the patient or legal representative depending on the patient’s medical condition) including cognitive deficits, headache, mRS and EQ-5D at 90 ±2 days after start of ventilation.

# **STATISTICAL METHODS**

## **Hypothesis**

The null hypothesis is that audio recordings of patients' relatives support weaning from mechanical ventilation and reduce weaning failure in patients with severe brain injury.

## **Estimation of Sample Size**

The sample size is estimated based on the hypothesis of ARPR achieving a 15% absolute weaning failure reduction with an event rate of 44% in the sham-control group, 80% power and 5% α-risk. To correct for dropouts and lost to follow-up, the sample size is increased by 10% resulting in 354 patients. An interim analysis is planned after inclusion of 50% of the calculated subjects.

## **Statistical methods**

Statistical analyses will be performed using the current version of SPSS and/or STATA. Results will be expressed as mean (± SD) or median (interquartile rage) or number of events (percentage). Categorial variables will be compared by the Pearsons χ2 test or the Fisher’s exact test, as appropriate. Ordinal variables and non-normally distributed continuous variables will be compared by the Mann–Whitney U-test, normally distributed continuous variables by the Anova test. Measure of effect will be absolute differences. Sensitivity analyses will be performed if substantial non-compliance occurs and to evaluate the effect of adjustment for potential intergroup differences in baseline characteristics measured by standardized mean differences. Subgroup analyses will be undertaken for age (≤60 years / >60 years), sex (women / men), level of consciousness before intubation (measured by the GCS: 3-4 / 5-12 / 13-15), type of disease (intracerebral hemorrhage / ischemic stroke / subarachnoid hemorrhage / other diseases), duration of ventilation prior to weaning initiation (≤6 days / >6 days), occurrence of tracheostomy (yes / no) and ICU-delirium (yes / no).

## **Analysis population**

All analyses will be done on the modified intention-to-treat. Randomized participants receiving at least 1 administration of study procedure will be included.

## **Missing data**

Dropout patients are defined as patients who withdraw consent or whom representatives withdraw consent or for whom the randomized intervention did not start. These patients will be replaced and not included in the analysis of primary and secondary outcomes.

# **QUALITY ASSURANCE AND AUDIT**

## **Insurance**

The study sponsor takes full responsibility for potential damages which participants may suffer in accordance with applicable legal provisions.

## **Study Data**

Study data will be collected by authorized study collaborators (delegated by the Chief Investigator) and recorded in the eCRF. Participant identification in the CRF will be through unique participant study numbers allocated at the time of randomization.

## **Record Retention**

All records, documents and the study database will be stored for ten years or for longer if required.

## **Monitoring and quality assurance of study procedures**

The Chief Investigator will carry out monitoring of study data as an ongoing activity to ensure the safety of study participants and integrity of the study data. Monitoring of study data includes confirmation of informed consent; source data verification; data storage and data transfer procedures; validation of data. The study database will be checked and verified against the source data. The DSMB will perform regular visits to ensure protocol compliance, quality of data collection and documentation.

Deviations from clinical trial protocol must be documented and appropriate corrective and preventative actions taken. A serious breach is defined as any deviation from the protocol or from the principles of GCP in connection with the Voice-Weaning study that has a significant effect on the safety or integrity of the subjects or the scientific value of the study. Any serious breaches of the study protocol or GCP should be immediately reported to the Chief Investigator. The Chief Investigator in consultation with the DSMB will take whatever immediate action is required to ensure wellbeing of participants.

## **Audits and Inspections**

There is no trial audit planned. However, in case of an audit, the study documentation and source data/documents will be accessible to auditors of the sponsor/inspectors of the REC and questions will be answered.

## **Confidentiality**

Medical information obtained as a result of study participation are confidential and disclosure to third parties is prohibited. Subject identification code numbers are used in the computer files for participant confidentiality.

# **PUBLICATION AND DISSEMINATION POLICY**

After study completion, the results of the VOICE-WEANING study will be presented at national and international congresses and submitted to peer-reviewed journals as scientific papers. Substantial contribution has to be made by all authors.

# **FUNDING**

## **Funding source**

The VOICE-WEANING trial is supported by a research grant (Alz/Iko – Sprü/2020) from the Johannes and Frieda Marohn Foundation, University of Erlangen, Germany.

## **Participants payments**

There is no payment to participants in this study.

# **REFERENCES**

1. Ely EW, Margolin R, Francis J, May L, Truman B, Dittus R*, et al.* Evaluation of delirium in critically ill patients: validation of the Confusion Assessment Method for the Intensive Care Unit (CAM-ICU). *Critical care medicine* 2001; 29: 1370-1379.

2. Boles JM, Bion J, Connors A, Herridge M, Marsh B, Melot C*, et al.* Weaning from mechanical ventilation. *The European respiratory journal* 2007; 29: 1033-1056.

3. Thomalla G, Simonsen CZ, Boutitie F, Andersen G, Berthezene Y, Cheng B*, et al.* MRI-Guided Thrombolysis for Stroke with Unknown Time of Onset. *The New England journal of medicine* 2018; 379: 611-622.

4. Hemphill JC, 3rd, Bonovich DC, Besmertis L, Manley GT, Johnston SC. The ICH score: a simple, reliable grading scale for intracerebral hemorrhage. *Stroke* 2001; 32: 891-897.

5. Welte TM, Gabriel M, Hopfengärtner R, Rampp S, Gollwitzer S, Lang JD*, et al.* Quantitative EEG may predict weaning failure in ventilated patients on the neurological intensive care unit. *Scientific reports* 2022; 12: 7293.

6. Mueller TM, Gollwitzer S, Hopfengärtner R, Rampp S, Lang JD, Stritzelberger J*, et al.* Alpha power decrease in quantitative EEG detects development of cerebral infarction after subarachnoid hemorrhage early. *Clinical neurophysiology : official journal of the International Federation of Clinical Neurophysiology* 2021; 132: 1283-1289.

7. Gollwitzer S, Groemer T, Rampp S, Hagge M, Olmes D, Huttner HB*, et al.* Early prediction of delayed cerebral ischemia in subarachnoid hemorrhage based on quantitative EEG: A prospective study in adults. *Clinical neurophysiology : official journal of the International Federation of Clinical Neurophysiology* 2015; 126: 1514-1523.

8. Hopfengärtner R, Kerling F, Bauer V, Stefan H. An efficient, robust and fast method for the offline detection of epileptic seizures in long-term scalp EEG recordings. *Clinical neurophysiology : official journal of the International Federation of Clinical Neurophysiology* 2007; 118: 2332-2343.

9. Wunsch H, Linde-Zwirble WT, Angus DC, Hartman ME, Milbrandt EB, Kahn JM. The epidemiology of mechanical ventilation use in the United States. *Critical care medicine* 2010; 38: 1947-1953.

10. Lahiri S, Mayer SA, Fink ME, Lord AS, Rosengart A, Mangat HS*, et al.* Mechanical Ventilation for Acute Stroke: A Multi-state Population-Based Study. *Neurocritical care* 2015; 23: 28-32.

11. Esteban A, Anzueto A, Frutos F, Alía I, Brochard L, Stewart TE*, et al.* Characteristics and outcomes in adult patients receiving mechanical ventilation: a 28-day international study. *Jama* 2002; 287: 345-355.

12. Pelosi P, Ferguson ND, Frutos-Vivar F, Anzueto A, Putensen C, Raymondos K*, et al.* Management and outcome of mechanically ventilated neurologic patients. *Critical care medicine* 2011; 39: 1482-1492.

13. Mancebo J. Weaning from mechanical ventilation. *The European respiratory journal* 1996; 9: 1923-1931.

14. Stevens JP, Howell MD. Liberating brain-injured patients from mechanical ventilation. learning from healthcare delivery science. *American journal of respiratory and critical care medicine* 2013; 188: 894-896.

15. Alex CG, Fahey PJ, Tobin MJ. What a Clinician Should Do When a Patient “Fights the Ventilator”. Berlin, Heidelberg: Springer Berlin Heidelberg; 2002. p. 100-117.

16. Sinderby C, Navalesi P, Beck J, Skrobik Y, Comtois N, Friberg S*, et al.* Neural control of mechanical ventilation in respiratory failure. *Nature medicine* 1999; 5: 1433-1436.

17. Coplin WM, Pierson DJ, Cooley KD, Newell DW, Rubenfeld GD. Implications of extubation delay in brain-injured patients meeting standard weaning criteria. *American journal of respiratory and critical care medicine* 2000; 161: 1530-1536.

18. Addressing The Nurse Shortage To Improve The Quality Of Patient Care. *Health Affairs* 2006; 25: 268-274.

19. Supady A, Curtis JR, Abrams D, Lorusso R, Bein T, Boldt J*, et al.* Allocating scarce intensive care resources during the COVID-19 pandemic: practical challenges to theoretical frameworks. *The Lancet Respiratory Medicine*.

20. White DB, Angus DC, Shields AM, Buddadhumaruk P, Pidro C, Paner C*, et al.* A Randomized Trial of a Family-Support Intervention in Intensive Care Units. *The New England journal of medicine* 2018; 378: 2365-2375.

21. Gorji MA, Araghiyansc F, Jafari H, Gorgi AM, Yazdani J. Effect of auditory stimulation on traumatic coma duration in intensive care unit of Medical Sciences University of Mazandarn, Iran. *Saudi J Anaesth* 2014; 8: 69-72.

22. Tavangar H, Shahriary-Kalantary M, Salimi T, Jarahzadeh M, Sarebanhassanabadi M. Effect of family members' voice on level of consciousness of comatose patients admitted to the intensive care unit: A single-blind randomized controlled trial. *Adv Biomed Res* 2015; 4: 106.

23. MacIntyre NR, Cook DJ, Ely EW, Jr., Epstein SK, Fink JB, Heffner JE*, et al.* Evidence-based guidelines for weaning and discontinuing ventilatory support: a collective task force facilitated by the American College of Chest Physicians; the American Association for Respiratory Care; and the American College of Critical Care Medicine. *Chest* 2001; 120: 375s-395s.

# **12. APPENDICES**

Not applicable.
